# Hematopoietic-Specific DARC Deficiency Is Associated with Adipose Tissue Inflammation and Impaired Glucose Tolerance During Diet-Induced Obesity in Mice

**DOI:** 10.3390/cells15181664

**Published:** 2026-09-15

**Authors:** Ghaith Aboud, Tyler W. Benson, Ragheb Harb, Praneet Veerapaneni, Guangwei Zhang, Samah Ahmadieh, Rishabh Agrawal, Charlotte Greenway, Hunter Sellers, Brandee Goo, David S. Kim, Mourad Ogbi, Stephen Cave, Mehek Sharma, Lingling Liu, Catherine C. Hedrick, Sabrina Robichaud, Hong Shi, Avirup Guha, Ryan A. Harris, Xiaoling Wang, David Stepp, Yun Lei, Quangsheng Du, Ha Won Kim, Xin-Yun Lu, Neal L. Weintraub

**Affiliations:** 1 Vascular Biology Center, Medical College of Georgia, Augusta University, 1460 Laney Walker Blvd, Augusta, GA 30912, USA; gaboud@augusta.edu (G.A.); bensonte@ucmail.uc.edu (T.W.B.); rharb@augusta.edu (R.H.); pveerapaneni@achehealth.edu (P.V.); zhang.guangwei2@mayo.edu (G.Z.); sahmadieh@augusta.edu (S.A.); rishabh.agrawal@ufl.edu (R.A.); charlotte.marie.mccrary@emory.edu (C.G.); hsellers@augusta.edu (H.S.); bgoo@emory.edu (B.G.); skim1@augusta.edu (D.S.K.); mogbi@augusta.edu (M.O.); scave@augusta.edu (S.C.); mesharma@augusta.edu (M.S.); lliu1@augusta.edu (L.L.); hoshi@augusta.edu (H.S.); aguha@augusta.edu (A.G.); dstepp@augusta.edu (D.S.); hkim3@augusta.edu (H.W.K.); 2 Immunology Center of Georgia, Augusta University, 1410 Laney Walker Blvd, Augusta, GA 30912, USA; lhedrick@augusta.edu (C.C.H.);; 3 Department of Medicine, Medical College of Georgia, Augusta University, 1460 Laney Walker Blvd, Augusta, GA 30912, USA; 4 Georgia Prevention Institute, Medical College of Georgia, Augusta University, 1499 Walton Wy, Augusta, GA 30912, USA; ryharris@augusta.edu (R.A.H.); xwang@augusta.edu (X.W.); 5 Department of Neuroscience and Regenerative Medicine, Medical College of Georgia, Augusta University, 1460 Laney Walker Blvd, Augusta, GA 30912, USA; ylei@augusta.edu (Y.L.); qdu@augusta.edu (Q.D.); xylu@augusta.edu (X.-Y.L.)

**Keywords:** ACKR1, DARC, glucose tolerance, hematopoietic cell, adipose tissue

## Abstract

**Highlights:**

- Hematopoietic-specific disruption of DARC is associated with adipose tissue inflammation and oxidative stress during obesity, leading to glucose intolerance.
- DARC expressed in adipocytes may regulate aspects of adipocyte morphology in vivo. However, deletion of DARC in mature adipocytes or endothelial cells does not alter glucose tolerance.

**Abstract:**

Objective: Adipose tissue inflammation in obesity promotes insulin resistance and metabolic disease. The Duffy Antigen Receptor for Chemokines (DARC), a non-signaling receptor expressed on erythrocytes and on other cell types, modulates inflammation by regulating chemokine levels. Gene variants that affect DARC expression on erythrocytes are common in people of African descent. Here, we disrupted DARC expression in hematopoietic cells and adipocytes to determine the impact on obesity. Methods: DARC floxed mice were bred with vav1-Cre mice to disrupt DARC expression in hematopoietic cells (DARC^Δhemato^), and with adipoq-Cre mice to disrupt DARC expression in adipocytes (DARC^Δadipo^). Mice were fed a chow diet (CD) versus a high-fat diet (HFD) and studied in vivo. In vitro studies were conducted on preadipocytes isolated from wild-type (WT) and global DARC knockout mice. Results: HFD-fed DARC^Δhemato^ mice exhibited impaired glucose tolerance compared with WT littermates, independent of weight gain. This was associated with increased adipose tissue inflammation and systemic oxidative stress. DARC^Δadipo^ mice exhibited similar glucose tolerance but had subtle differences in adipose depot mass and adipocyte size compared with WT littermates. Deletion of DARC in preadipocytes had no impact on adipogenic differentiation or lipid accumulation in vitro. Similarly, endothelial-specific DARC KO mice also showed no differences in glucose tolerance compared with WT. Conclusions: DARC disruption in hematopoietic cells is associated with adipose tissue inflammation and alters glucose homeostasis during diet-induced obesity, accompanied by reduced circulating levels of several DARC-binding chemokines. The relevance of these findings to human DARC genetic variants and obesity-related metabolic disease warrants further investigation.

## 1. Introduction

Obesity is characterized by chronic low-grade adipose tissue inflammation associated with increased production of pro-inflammatory cytokines and chemokines. Certain adipocytokines and chemokines, such as tumor necrosis factor alpha (TNFα) [1] and monocyte chemoattractant protein-1 (MCP-1) [2], have been causally linked to the development of insulin resistance, a key feature of obesity-related metabolic disease and type 2 diabetes.

Chemokines bind not only to their respective signaling receptors, but also to several non-signaling receptors, the most noteworthy of which is the atypical chemokine receptor 1 (ACKR1), also known as Duffy antigen receptor for chemokines (DARC). In humans, DARC is expressed primarily on erythrocytes and capillary or post-capillary endothelial cells, where it is thought to sequester chemokines and/or regulate their local concentration at sites of inflammation. DARC is promiscuous and can bind to a variety of C-C and C-X-C motif chemokines [3]. Indeed, loss of functional DARC correlated with decreased levels of DARC-bound chemokines in the serum in both humans and mice [4,5]. DARC has a particularly strong binding affinity for C-C motif chemokine ligand 2 (CCL2)/MCP-1, and loss of DARC enhances MCP-1-induced monocyte mobilization and lipopolysaccharide-induced inflammation [3], suggesting an important role for DARC’s ability to sequester chemokines in controlling inflammation.

Three main alleles of the DARC gene are present in the human population: FYB, FYA and FYO, the prevalence of which varies by region and ancestry [6,7]. FYA is the most common allele globally and is most prevalent in those of Asian descent; FYB predominates in those of European descent, while FYO is most prevalent in those of African descent. The FYA allele is globally hypofunctional, while the FYO allele is associated with selective loss of DARC expression on erythrocytes and is thought to result from selection pressure imposed by *Plasmodium vivax* malaria. *P. vivax* engages DARC to penetrate erythrocytes, and the absence of DARC expression on erythrocytes confers resistance to the parasite [8]. African Americans exhibit heightened inflammation and are at increased risk of obesity-related metabolic disease compared with Caucasians [9]. While these latter findings may largely be explained by environmental factors such as diet, exercise, etc., insulin sensitivity is also reportedly lower in non-obese African Americans [10,11], suggesting that ancestral factors such as DARC genotype may also be contributory.

We previously reported that global deletion of DARC in mice fed a high-fat diet (HFD) resulted in increased adipose tissue inflammation and insulin resistance [12]. Interestingly, non-obese DARC knockout (KO) mice fed a standard chow diet (CD) also exhibited impaired glucose tolerance compared with wild-type mice. This raised the possibility that DARC can regulate insulin-induced glucose uptake in non-obese states, in the absence of upregulated chemokine concentrations, and independent of its expression in cells of hematopoietic origin. Given that DARC is expressed across multiple cell types, including hematopoietic cells, adipocytes, and endothelial cells, an important knowledge gap is whether DARC regulates obesity-associated glucose intolerance through specific cellular compartments. Here, we generated a DARC-floxed mouse to determine whether disruption of DARC expression in hematopoietic cells exacerbates obesity-related metabolic disease. We also examined DARC expression in insulin-sensitive tissues and unexpectedly detected a high level of expression in adipose tissues. Hence, we also tested the role of DARC expression in mature adipocytes in diet-induced obesity.

## 2. Methods

### 2.1. Data Availability

Datasets analyzed in this study are available from the corresponding authors upon reasonable request.

### 2.2. Animals

Global DARC knockout mice in the C57BL/6J background were obtained from Jackson Laboratories. DARC floxed mice were generated by flanking the loxP site in the promoter region and exon 2 of DARC (Supplementary Figure S1A). To generate hematopoietic-, adipocyte-, or endothelial cell (EC)-specific DARC KO mice, DARC floxed (DARC^flox/flox^) mice in the C57BL/6 background were crossed with Vav-1-Cre mice, adiponectin-Cre mice, or VE-Cadherin-Cre mice (purchased from Jackson Laboratories, Augusta, ME, USA, C57BL/6 background), respectively. 8-week-old male mice were maintained on CD (Harlan Teklad, LM-485, New Brunswick, NJ, USA) or switched to HFD (Research Diet, D12492, 60% calories from fat, 20% calories from carbohydrate, 20% calories from protein, New Brunswick, NJ, USA) for up to 40 weeks. Food and water were provided ad libitum. Mice were weighed weekly. Following 40 weeks of HFD feeding, mice were sacrificed and tissues were harvested and weighed. Mice were euthanized with intraperitoneal pentobarbital 150 mg/kg, inhaled anesthesia (isoflurane) followed by carbon dioxide (CO_2_) narcosis and cervical dislocation or bilateral thoracotomy, in accordance with AVMA Panel 2007 recommendations and institutional IACUC guidelines. All mice were randomized to different treatment groups to minimize experimental variability. Experiments were performed independently and the resulting data were combined for analysis. Animals that became severely ill or died during the study (one mouse in each experimental group in the cohort of WT and adipocyte-specific DARC KO mice) was excluded from the final analysis. All animal studies were conducted using a protocol approved by the Institutional Animal Care and Use Committee of Augusta University following appropriate guidelines.

### 2.3. Body Composition Measurements

Fat and lean mass were measured in approximately 40-week-old mice using nuclear magnetic resonance (NMR) spectroscopy (Bruker Minispec LF90II, Billerica, MA, USA) and normalized to total body weight as previously reported [12,13].

### 2.4. Glucose Tolerance Test (GTT)

At 35 weeks (hematopoietic-specific DARC KO mice) and 26 weeks (adipocyte-specific DARC KO mice) of CD or HFD feeding, mice were fasted for 12 h followed by intraperitoneal injection of glucose at 2 g/kg body weight. Glucose concentrations were measured via the tail vein at baseline and every 10 min up to 2 h following glucose injection as previously described [12,13].

### 2.5. Insulin Tolerance Test (ITT)

At approximately 37 weeks (hematopoietic-specific DARC KO mice) and 28 weeks (adipocyte-specific DARC KO mice) of CD or HFD feeding, mice were fasted for 6 h followed by intraperitoneal injection with regular insulin (Humulin) at 0.75 units/kg body weight. Glucose concentrations were measured at baseline and every 10 min up to 90 min as described above [12,13].

### 2.6. Enzyme-Linked Immunosorbent Assay (ELISA)

Whole blood collected at sacrifice was incubated with 50 U/mL of heparin for 30 min with gentle rocking and separated by centrifugation at 3000× *g* for 10 min at 4 °C. Plasma levels of MCP-1 (R&D Systems, Minneapolis, MN, USA, sensitivity = 0.666 pg/mL, intra-assay precision coefficient of variation (%CV) = 2.5, inter-assay precision %CV = 5.1), IL-6 (R&D Systems, sensitivity = 7.8 pg/mL, intra-assay precision coefficient of variation (%CV) = 3.5, inter-assay precision %CV = 7.6), TNFα (R&D Systems, sensitivity = 7.21 pg/mL, intra-assay precision %CV = 3.1, inter-assay precision %CV = 8.0), insulin (R&D Systems, sensitivity = 1.88 pg/mL, intra-assay precision %CV = 5.6, inter-assay precision %CV = 5.5), 8-isoprostane (AFG Scientific, Arlington Heights, IL, USA, sensitivity = 9.375 pg/mL, intra-assay precision %CV < 8, inter-assay precision %CV < 10), 4-hydroxy-2-nonenal (HNE, Cell Biolabs, San Diego, CA, USA, limits of quantification and CV% are not available) and aspartate aminotransaminase (AST, Abcam, Waltham, MA, USA, sensitivity = 39 pg/mL, intra-assay precision %CV = 2.2, inter-assay precision %CV = 3.2) were quantified using commercially available ELISA kits according to the manufacturer’s protocol.

### 2.7. Quantitative PCR

Total RNA was extracted from visceral fat, subcutaneous fat, brown fat, skeletal muscle, liver, spleen, kidney, pancreas, heart, lung, brain and bone marrow with Qiazol and processed using the RNeasy Lipid Tissue Mini Kit (Qiagen, Germantown, MD, USA). Concentration and quality were verified by NanoDrop measurements (260/280 and 260/230). RT-PCR quantification of mRNA levels was performed using SYBR Green qRT-PCR kits (Agilent Technologies, ABM, Santa Clara, CA, USA) and the StepOnePlus Real-Time PCR System (Applied Biosystems, Waltham, MA, USA). Reaction conditions were 95 °C for 15 s followed by 60 °C for 1 min for 40 cycles. Primer sequences are shown in Supplementary Table S1. Fold change was calculated by the ΔΔCt method [12,13,14].

### 2.8. Western Blot

Protein was extracted from adipose tissues using a Tissue-Tearer (BioSpec, Bartlesville, OK, USA) in RIPA buffer with protease inhibitors, followed by centrifugation, separation by SDS-PAGE, transfer to a nitrocellulose membrane and probing with the appropriate antibodies. Blots were developed with an ECL system. Primary antibodies used were MCP-1 (Cell Signaling, #2029S, Danvers, MA, USA), GAPDH (Invitrogen, #AM4300, Waltham, MA, USA), phospho-Akt-Ser473 (Cell Signaling, #9271S) and total Akt (Cell Signaling, #9272). Anti-rabbit IgG was obtained from Cell Signaling (#7071S) and anti-mouse IgG was purchased from Cytiva (#NA931V, Marlborough, MA, USA). Primary and secondary antibodies were used at dilutions of 1:1000 and 1:20,000, respectively. Quantification of the Western blot was conducted by densitometric analysis using ImageJ software (v1.54p).

### 2.9. Primary Culture and Differentiation of Preadipocytes

Subcutaneous (inguinal) and visceral (epididymal) adipose tissue was isolated from 18–22-week-old WT and global DARC KO mice. Our in vivo studies were conducted over an extended period (up to 48 weeks on HFD), and GTT/ITT measurements were obtained from mice aged 27–38 weeks. To maintain consistency with these experimental conditions, we isolated primary preadipocytes from mice of comparable age (18–22 weeks) rather than from younger animals (e.g., 8–10 weeks). Briefly, following isolation, adipose tissue was thoroughly minced, digested for 1 h with collagenase type I (Worthington Biochemical, Lakewood, NJ, USA), and then passed through a 100 µm filter to generate a single-cell suspension as previously described [15]. Floating adipocytes were separated by centrifugation and the remaining cell pellet, consisting of stromovascular cells, was passed through a 40 µm filter, creating a preadipocyte-rich single-cell suspension. Next, cells were plated on a 10 cm dish and grown in preadipocyte growth medium (Cell Applications, San Diego, CA, USA) as previously described [14]. The cells were expanded for 2–4 passages in culture and then stimulated to differentiate using adipocyte differentiation medium (Cell Applications) for 14 days.

### 2.10. Measurement of Insulin-Stimulated Glucose Uptake In Vitro

Preadipocytes isolated from subcutaneous adipose tissue from WT and global DARC KO mice were differentiated into mature adipocytes as described above. Insulin (0.1 and 1 µg/mL) stimulated glucose uptake was measured using a commercially available Glucose Uptake-Glo Assay (Promega, Madison, WI, USA) according to the manufacturer’s instructions. The Glucose Uptake-Glo™ Assay is a plate-based, homogeneous bioluminescent method for measuring glucose uptake in cells, based on the detection of 2-deoxyglucose-6-phosphate (2DG6P).

### 2.11. Histology, Quantification of Hepatic Steatosis and Adipocyte Size

Liver and adipose tissues were fixed in 10% formalin, dehydrated in ethanol, transferred to xylene solution and embedded in paraffin. Tissue sections were stained with hematoxylin and eosin (H&E). Antibodies for F4/80 (Sigma-Aldrich, St. Louis, MO, USA) and DARC (Abcam) were used in conjunction with HistoMouse-SP kit (Invitrogen) or DAB Substrate kits (Vector Labs, Newark, CA, USA). Neutral lipids, stained with Oil Red O (ORO), were measured as previously described [16]. Briefly, fixed tissue sections or adipocytes were incubated with ORO working solution (3 parts 0.4% ORO stock solution: 2 parts ddH2O) for 10 min, and then washed with ddH_2_O and imaged under light microscopy. ORO in liver tissues and differentiated adipocytes was quantified using ImageJ software and spectrometry (optical density, 510 nm), respectively, as previously reported [14,15]. Adipocyte size and count were quantified using the Adiposoft plugin for Fiji as previously described [13].

### 2.12. Profiling of Pro-Inflammatory Cytokines

Mouse plasma cytokine profiling was performed at the Georgia Cancer Center Immune Monitoring Shared Resources at Augusta University using a Luminex 200 instrument (Augstin, TX, USA) and Mouse Inflammation 13-Plex Panel FbBA228ser (BioLegend, San Diego, CA, USA) to quantify key pro-inflammatory and anti-inflammatory cytokines as previously described [17].

### 2.13. Flow Cytometry

Blood was collected in EDTA-coated tubes by cardiac puncture. Red blood cells were lysed using BD Pharm Lyse lysing buffer (BD Biosciences, 555899) for 10 min and then resuspended in FACS buffer (2% FBS, 1 mM EDTA, 0.1% Sodium Azide in PBS). Monocyte, neutrophil, dendritic cell and natural killer (NK) cell frequencies were assessed by staining with the following antibodies: CD11b^+^ Ly6G^+^ (neutrophils), CD11c^+^ I-A/I-E^+^ (DCs), CD11b^+^ CD115^+^ (monocytes) and NK1.1^+^ CD3e^−^ (NK cells). A viability dye (Live/Dead Fixable Yellow, Invitrogen; 1:800 dilution) was used in all experiments. Data were acquired on a Cytek Aurora five-laser spectral flow cytometer (Cytek Biosciences, Fremont, CA, USA) and analyzed using FlowJo software (v10, BD Biosciences, San Jose, CA, USA).

### 2.14. Statistics

Generation of figures and statistical analysis were performed in GraphPad Prism (software version 10.6.1, Boston, MA, USA). Data are expressed as mean ± SEM. Shapiro-Wilk test was used to test normality. A Student’s *t*-test was used to analyze differences between two groups, while two-way ANOVA followed by a Bonferroni post-hoc analysis was used to compare differences between groups of three or more. For GTT and ITT, blood glucose measurements obtained repeatedly from the same animals over time were analyzed using two-way repeated-measures ANOVA, with genotype and time as factors. Genotype x time interactions were evaluated, followed by appropriate multiple-comparisons tests. The area under the curve (AUC) for the GTT and the glucose disappearance rate (KITT) for the ITT were calculated and analyzed separately as quantitative measures of glucose tolerance and insulin sensitivity, respectively. A *p*-value less than or equal to 0.05 was considered statistically significant.

## 3. Results

### 3.1. Hematopoietic-Specific Disruption of DARC Altered Glucose Tolerance Without Affecting Body Weight Gain During HFD Feeding

**Figure 1:**
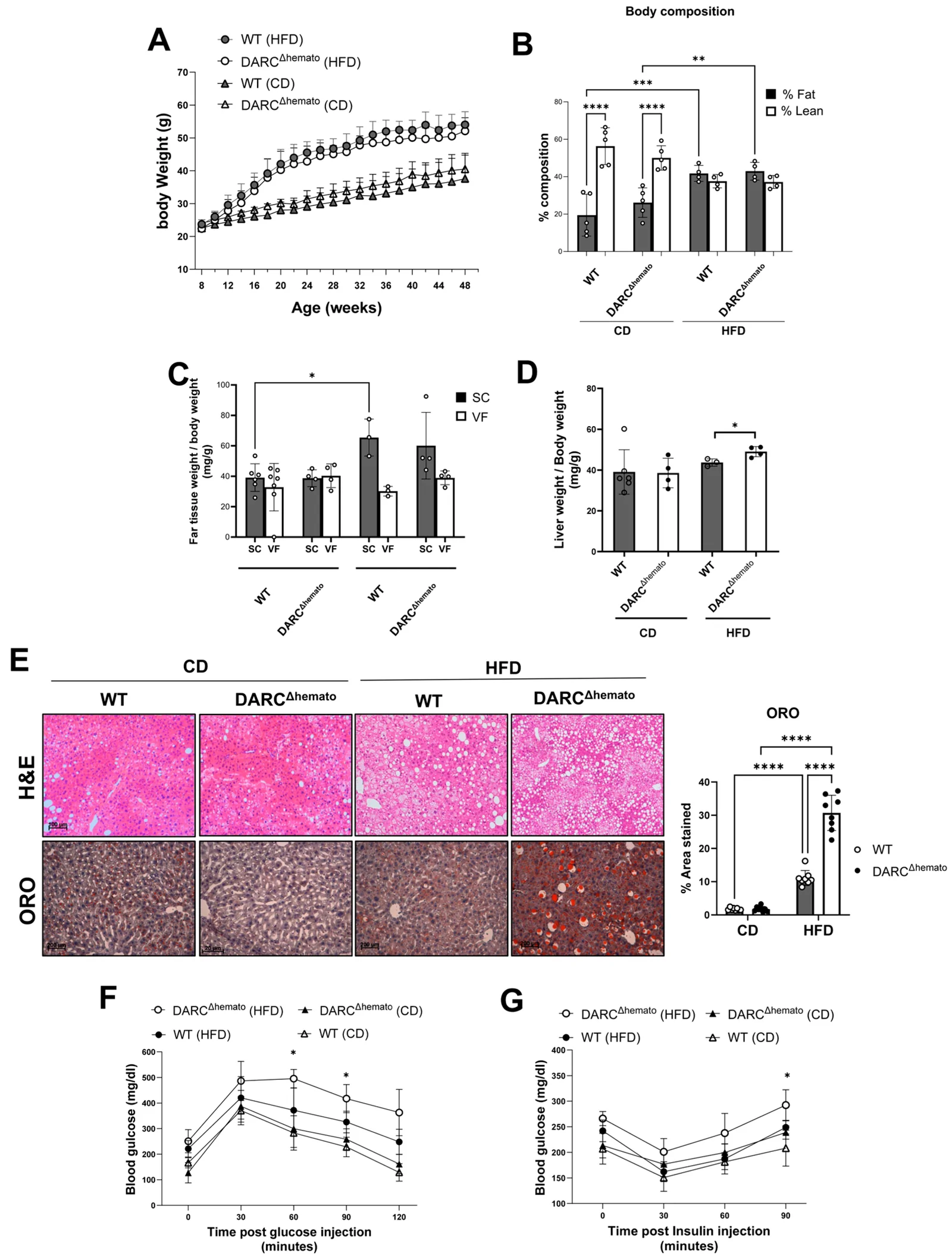
Hematopoietic-specific deletion of DARC impaired glucose tolerance without affecting body weight gain on HFD. (**A**) Growth curves of DARC^Δhemato^ and WT mice fed an HFD or CD (*n* = 8). (**B**) Whole-body composition (fat and lean mass) measured by NMR (n = 5). (**C**) Adipose tissue weight (SC and VF) normalized to body weight (n = 4). (**D**) Liver weight normalized to body weight (n = 4). (**E**) Representative H&E and ORO staining images of liver tissues and ORO quantification. (**F**) Glucose tolerance test after 38 weeks of CD or HFD (n = 5). (**G**) Insulin tolerance test after 40 weeks of CD or HFD (n = 5). * *p* < 0.05, ** *p* < 0.01, *** *p* < 0.001, **** *p* < 0.0001.

To delete DARC from hematopoietic cells, we bred Vav1-Cre mice with DARC floxed mice to create hematopoietic-specific DARC KO (DARC^Δhemato^) mice (Supplementary Figure S1A). Validation of hematopoietic-specific DARC deletion is shown in Supplementary Figure S1B. DARC mRNA expression was markedly reduced in bone marrow and spleen, but not in adipose tissue or brain, of DARC^Δhemato^ compared with wild-type (WT) mice (Supplementary Figure S1C). There was significant, but equivalent, weight gain in DARC^Δhemato^ and WT mice on HFD compared with mice maintained on CD (Figure 1A). Body composition by NMR spectroscopy showed a trend towards greater fat mass and smaller lean mass in the CD-fed DARC^Δhemato^ mice compared with WT littermates; however, these differences were not significant (Figure 1B). These trends were abolished by HFD feeding, during which both DARC^Δhemato^ and WT mice exhibited similar increases in fat mass and reductions in lean mass (Figure 1B). Adipose tissue weights (normalized to body weight) were similar in the CD-fed DARC^Δhemato^ mice compared with WT littermates, in both subcutaneous (SQ) and visceral fat (VF) tissue (Figure 1C). With HFD feeding, adipose tissue growth was primarily restricted to subcutaneous as opposed to visceral fat in both groups of mice. DARC^Δhemato^ mice developed more liver enlargement and steatosis, as demonstrated by H&E and ORO staining, during HFD feeding compared with WT mice (Figure 1D,E). However, we did not observe significant differences in plasma levels of aspartate aminotransferase (AST, Supplementary Figure S2) or lipids (cholesterol and triglycerides, Supplementary Figure S3) between WT and DARC^Δhemato^ mice. Glucose tolerance was similar in CD-fed WT and DARC^Δhemato^ mice (Figure 1F). Glucose concentrations tended to be higher in the HFD-fed DARC^Δhemato^ mice at all time points, reaching statistical significance at the 60 min and 90 min post-glucose injection time points (Figure 1F and Figure S4A). The HFD-fed DARC^Δhemato^ mice also had higher circulating blood glucose after insulin injection compared with WT littermates, with the difference reaching significance at the 90 min time point (Figure 1G and Figure S4A). Consistent with these findings, analysis of the glucose excursion demonstrated a significant increase in GTT AUC in HFD-fed DARC^Δhemato^ mice compared with WT mice (*p* = 0.0272). In contrast, the KITT derived from the ITT was not significantly different between genotypes (*p* = 0.5165), although blood glucose remained higher in DARC^Δhemato^ mice at the 90-min time point. Plasma insulin levels did not differ between WT and DARC^Δhemato^ mice (Supplementary Figure S5). These findings are consistent with our previous report demonstrating comparable beta cell abundance in pancreatic tissue from WT and global DARC KO mice [12]. Together, these results indicate that hematopoietic DARC deficiency is associated with impaired glucose tolerance during HFD feeding, without evidence of impaired insulin production. In addition, there was no significant difference in phosphorylation of Akt in adipose tissues, skeletal muscles and liver (Supplementary Figure S6). This may be because Akt phosphorylation is highly dynamic and transient, and a single time-point measurement may not capture differences in insulin signaling. In addition, the altered glucose tolerance observed in the GTT and ITT may involve Akt-independent mechanisms or alterations downstream of Akt. Interestingly, DARC^Δhemato^ mice exhibited trends toward increased circulating leukocytes, including monocytes and neutrophils (Supplementary Figure S7), as well as elevated levels of inflammatory cytokines such as IFNs and TNFα (Supplementary Figure S8). On the other hand, plasma levels of DARC-binding chemokines, including CCL2, CCL5, CCL11, and CXCL1 were markedly reduced (Supplementary Figure S9), suggesting that loss of DARC is associated with an altered systemic distribution of DARC-binding chemokines. This pattern is consistent with impaired DARC-mediated chemokine buffering, although alternative mechanisms affecting chemokine production, retention, degradation, or clearance cannot be excluded.

### 3.2. Increased Adipose Tissue Inflammation in HFD-Fed DARC^Δhemato^ Mice

**Figure 2:**
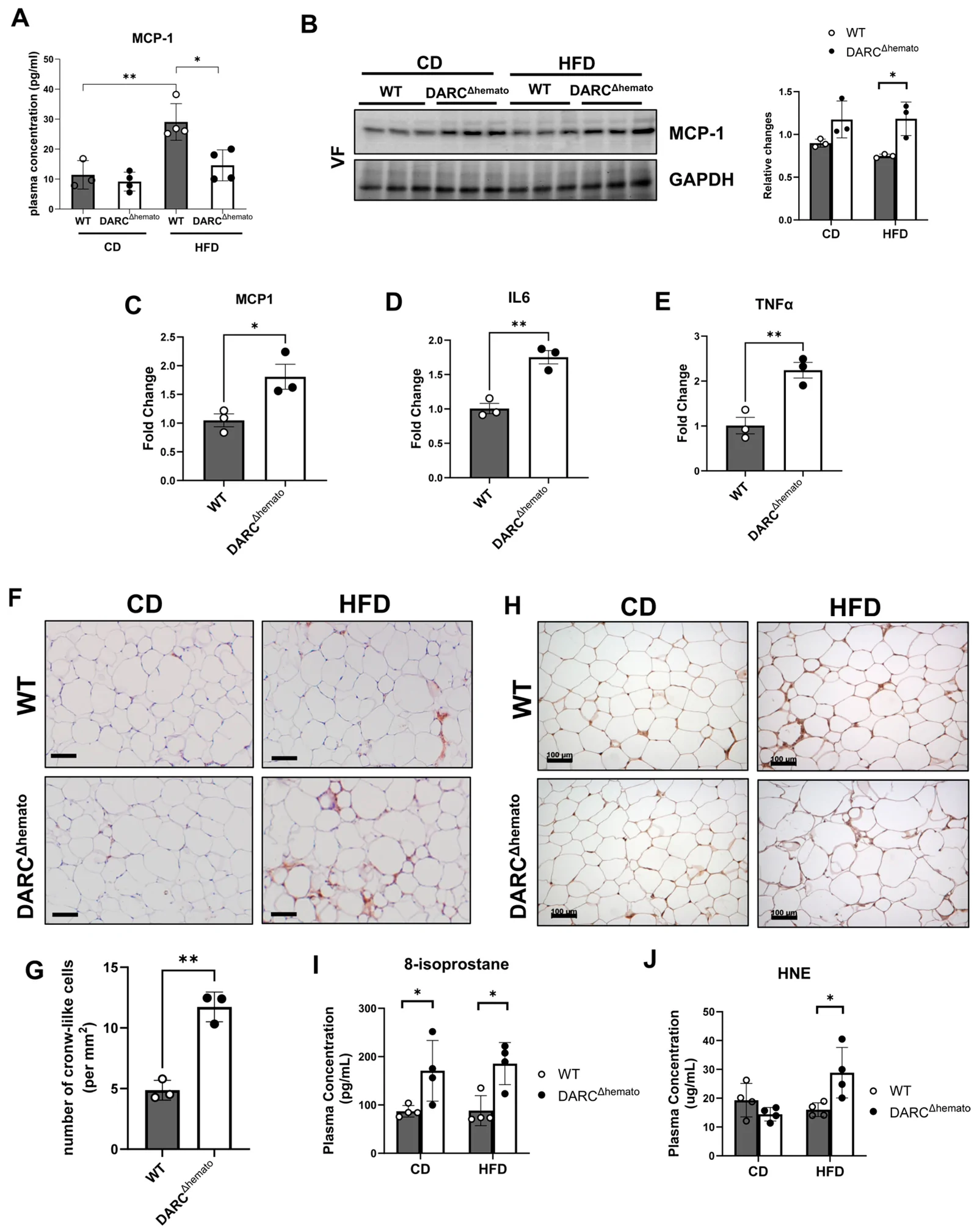
Increased adipose tissue inflammation and oxidative stress markers in circulation of HFD-fed DARC^Δhemato^ mice. (**A**–**C**) Plasma levels of MCP-1 (**A**) measured by ELISA following treatment of whole blood with heparin from mice on CD or HFD for 40 weeks (*n* = 3–4). (**B**) Protein expression of MCP-1 in VF (*n* = 3). (**C**–**E**) mRNA expression levels of MCP-1 (**C**), IL-6 (**D**), and TNFα (**E**) in visceral adipose tissue (*n* = 3). (**F**,**G**) Representative images (**F**) of F4/80 positive macrophage immunostaining in VF with quantification of crown-like structures (**G**) (*n* = 3). Scale bar = 100 µm. (**H**) Representative images of DARC staining in VF. Scale bar = 100 µm. (**I**,**J**) Plasma levels of 8-isoprostane (**I**) and HNE (**J**). * *p* < 0.05, ** *p* < 0.01.

Next, we measured the levels of selected chemokines and cytokines in the plasma and in visceral adipose tissues, which are more strongly associated with glucose intolerance and diabetes than subcutaneous adipose tissue [18]. Levels of MCP-1, a strong DARC-binding chemokine reported to promote adipose inflammation, rose significantly in the plasma of the WT mice with HFD feeding. However, the DARC^Δhemato^ mice tended to exhibit lower plasma MCP-1 levels at baseline, and no significant increase was noticed following HFD feeding (Figure 2A). In contrast, MCP-1 protein levels were higher in visceral fat of HFD-fed DARC^Δhemato^ mice compared with WT mice (Figure 2B). Additionally, mRNA levels of *Mcp-1* (Figure 2C), *Il-6* (Figure 2D), *Tnfα* (Figure 2E) and other chemokines, including CCL5 and CXCL1 (Supplementary Figure S10) were increased in visceral fat of DARC^Δhemato^ mice on HFD compared with WT mice.

Adipose tissue immunostaining demonstrated few infiltrating F4/80 positive macrophages in VF of CD-fed WT or DARC^Δhemato^ mice; however, following HFD feeding, there was an increase in macrophage staining in both groups, with significantly more crown-like structures in HFD-fed DARC^Δhemato^ mice, consistent with increased adipose tissue inflammation (Figure 2F,G). Increased adipose tissue inflammation was further supported by changes in the expression of selected pro- and anti-inflammatory macrophage-associated genes, including increased Nox2 and Ptgs2 and decreased Arg1 and Retnla (Supplementary Figure S11). Despite the increased adipose tissue inflammation, we detected no difference in adipocyte number or size between WT and DARC^Δhemato^ mice fed an HFD (Supplementary Figure S12). Moreover, there was no significant difference in DARC expression in VF between WT and DARC^Δhemato^ mice fed either a CD or HFD (Figure 2H and Figure S13). Adipose tissue inflammation and systemic oxidative stress are closely linked in obesity [19]. Accordingly, plasma levels of 8-isoprostane (Figure 2I) and HNE, markers of oxidative stress (Figure 2J), were significantly increased in HFD-fed DARC^Δhemato^ mice compared with WT mice.

### 3.3. Loss of DARC Expression Had No Impact on Adipogenic Differentiation and Glucose Uptake

**Figure 3:**
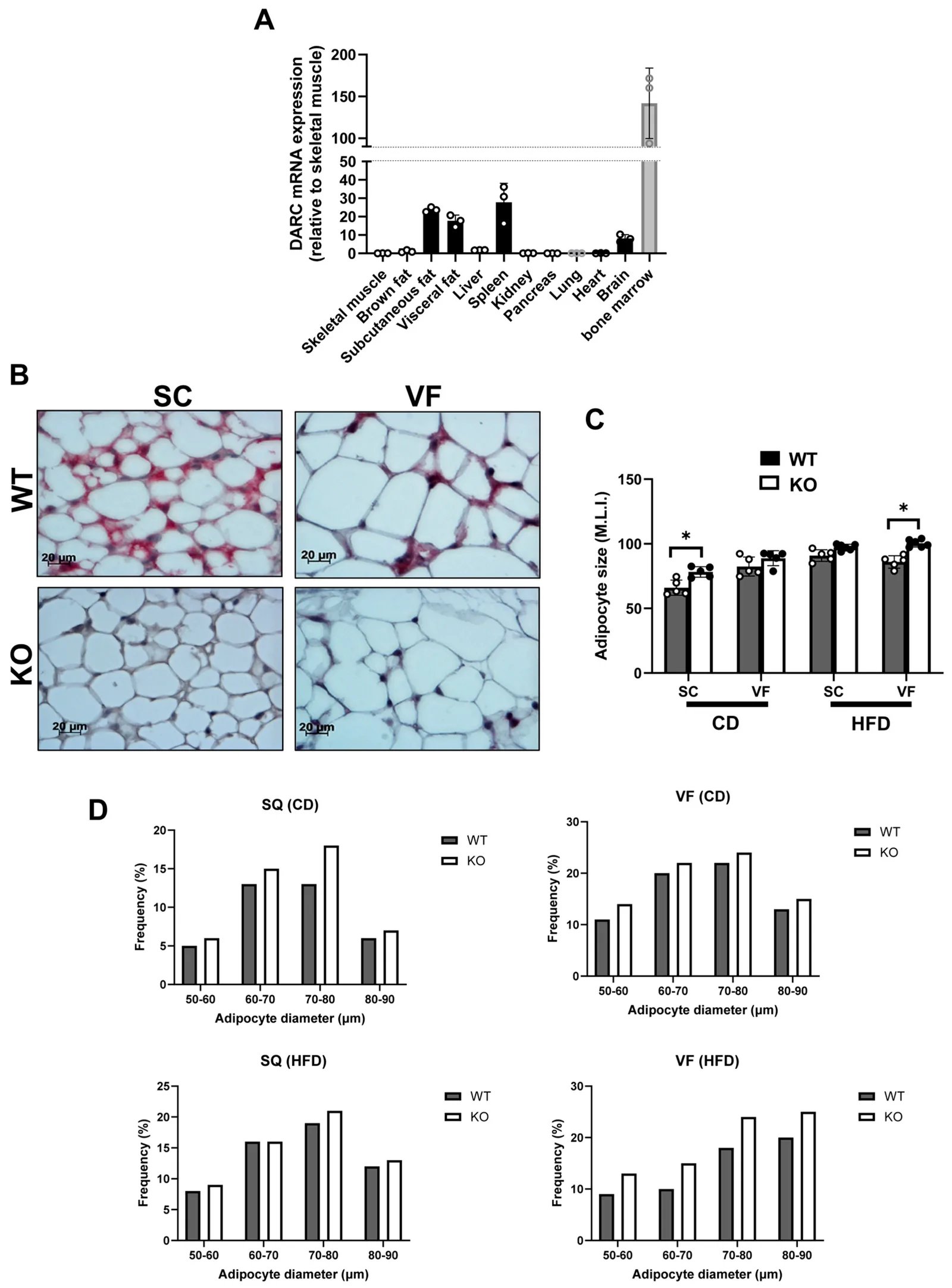
DARC is highly expressed in adipose tissues, spleen and bone marrow. (**A**) DARC mRNA expression measured by qRT-PCR in various tissues, normalized to brown fat (*n* = 3). (**B**) Representative images of DARC immunostaining in SC and VF. (**C**) Adipocyte size measured by mean linear intercept (M.L.I.) in SC and VF in WT and DARC KO mice (*n* = 5). * *p* < 0.05. (**D**) Frequency distribution of adipocyte size.

Since we previously reported that global DARC KO mice fed a CD exhibited impaired glucose tolerance, we examined DARC mRNA expression in various tissues and organs, specifically those that are highly insulin sensitive. As expected, in CD-fed mice, we detected a high level of DARC expression in bone marrow, and to a lesser extent in the spleen (Figure 3A). DARC mRNA expression was minimal in the pancreas (Figure 3A). Interestingly, we also detected significant DARC mRNA expression in both subcutaneous and visceral adipose tissues relative to other insulin-sensitive tissues (Figure 3A). DARC protein expression was detected in both subcutaneous and visceral adipose tissue of WT mice fed a CD using immunohistochemistry (adipose tissue collected from global DARC KO mice served as a negative control, Figure 3B). Interestingly, CD-fed global DARC KO mice had significantly larger subcutaneous adipocytes, and there was a trend toward larger visceral adipocytes, compared with WT mice (Figure 3B,C). In contrast, in HFD-fed mice, global DARC gene deletion led to enlarged visceral adipocytes, while the subcutaneous adipocytes were similar in size (Figure 3B,C).

**Figure 4:**
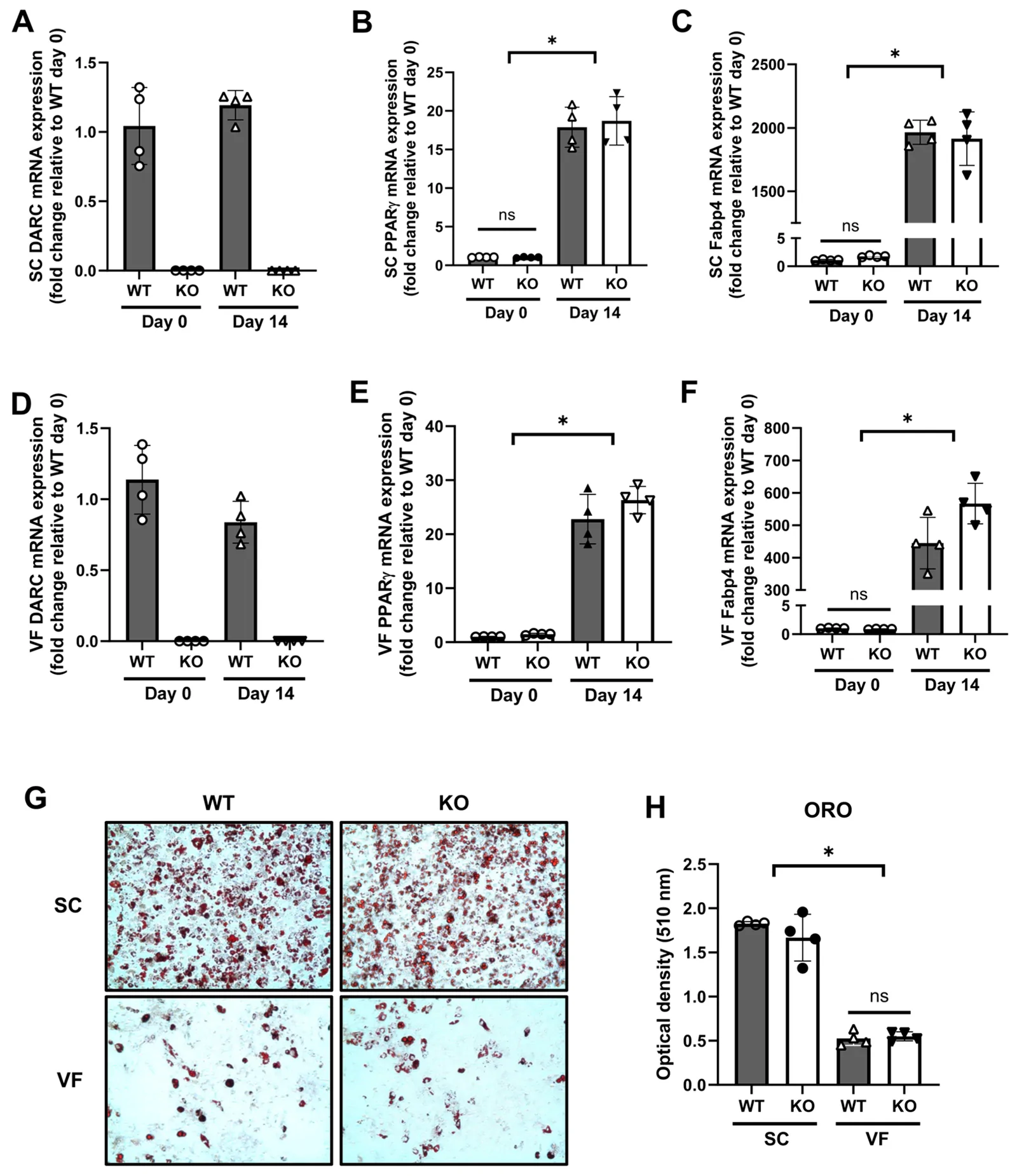
Loss of DARC does not impair adipogenesis or lipid accumulation in vitro. (**A**) DARC mRNA expression (**A**,**D**), Pparγ (**B**,**E**) and Fabp4 (**C**,**F**) before (day 0) and after adipogenic differentiation (day 14) (*n* = 4). Preadipocytes were isolated from SC (**A**–**C**) and VF (**D**–**F**) and differentiated into mature adipocytes in vitro. (**G**,**H**) Representative images of ORO staining at 14 days of adipogenic differentiation (**G**) and quantification (**H**) (*n* = 4). * *p* < 0.05, ns = not significant.

Given the increase in size of adipocytes and the reduced glucose intolerance reported previously in global DARC KO mice, we investigated the impact of deletion of DARC on adipogenic differentiation in vitro. To this end, preadipocytes were isolated from subcutaneous and visceral adipose tissues of WT and global DARC KO mice by collagenase digestion. Preadipocytes were then cultured and differentiated into mature adipocytes following an established adipogenic differentiation protocol. Using qRT-PCR, we measured the expression level of DARC and adipogenic markers *Pparγ* and *Fabp4* at day 0 and day 14 of differentiation. As expected, DARC was undetectable by qRT-PCR in cells isolated from DARC KO mice (Figure 4A,D). In cells isolated from both subcutaneous and visceral adipose depots of WT mice, DARC expression was similar at day 0 and day 14 of adipogenic differentiation (Figure 4A,D). Additionally, while there was a significant increase in *Pparγ* (Figure 4B,E), *Fabp4* (Figure 4C,F), adiponectin (Supplementary Figure S14A,C) and C/EBPα (Supplementary Figure S14B,D) at day 14 compared with day 0, we found no difference between WT and DARC KO cells isolated from either subcutaneous or visceral adipose depots (Figure 4B,C,E,F and Figure S14). As expected, differentiation of both subcutaneous and visceral preadipocytes was associated with lipid accumulation (ORO staining), the capacity of which was higher in subcutaneous compared with visceral cells (Figure 4G,H). However, loss of DARC expression had no impact on lipid accrual in either subcutaneous or visceral cells (Figure 4G,H). Preadipocytes derived from visceral fat are known to exhibit lower adipogenic potential compared with those from subcutaneous adipose tissue [20], and this impairment is further exacerbated under HFD conditions [14]. Consistent with these findings, our results show reduced adipogenic differentiation in visceral fat-derived preadipocytes. These results suggest that, while DARC expression is maintained during adipogenic differentiation, the capacity for differentiation is not altered by loss of DARC.

We next measured insulin-dependent glucose uptake using preadipocytes from subcutaneous adipose tissue differentiated into mature adipocytes for 14 days. In vitro glucose uptake was similar in differentiated preadipocytes from WT versus global DARC KO mice (Supplementary Figure S15A). AKT phosphorylation, an indicator of insulin signaling, tended to be increased in subcutaneous adipocytes from DARC KO mice compared with WT mice at baseline and following insulin stimulation (Supplementary Figure S15B). However, the mechanistic relationship between DARC and pAKT signaling remains unclear. These findings suggest that genetic DARC deletion does not impair insulin-stimulated glucose uptake or AKT phosphorylation in adipocytes in vitro.

### 3.4. Adipocyte-Specific Deletion of DARC Was Not Associated with Impaired Glucose Tolerance During HFD Feeding

**Figure 5:**
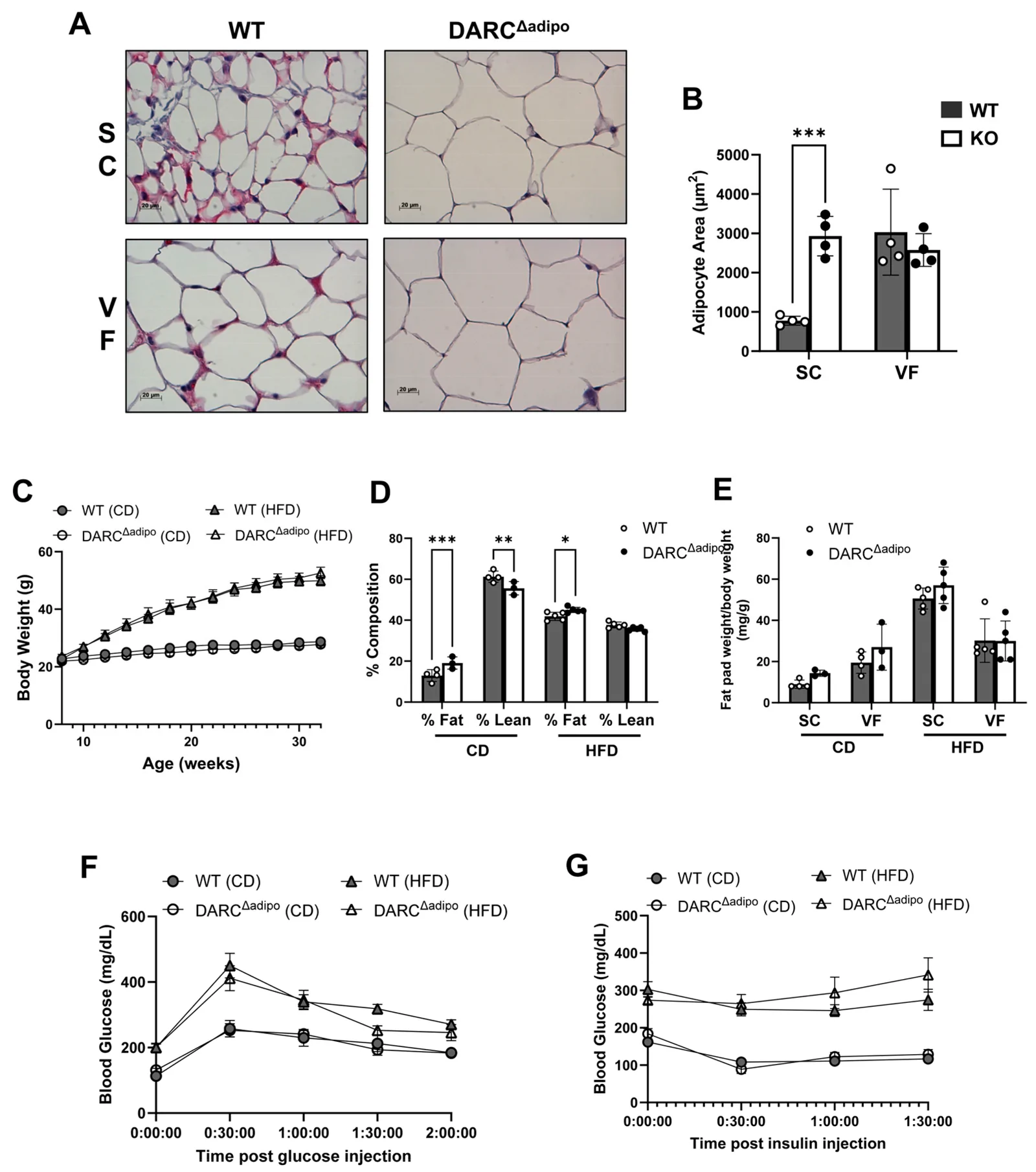
Deletion of adipocyte DARC did not affect body weight gain or glucose tolerance. (**A**,**B**) Adipocyte size measured in SC and VF of DARC^ΔAdipo^ and littermate WT mice (*n* = 4). (**C**) Growth curves of DARC^ΔAdipo^ and WT mice fed either HFD or CD (n = 6). (**D**) Whole-body composition (lean and fat mass measured by NMR (*n* = 3–5). (**E**) SC and VF pad weight normalized to body weight (*n* = 3–5). (**F**,**G**) Glucose tolerance after 20 weeks HFD (**F**) and insulin tolerance testing after 21 weeks HFD (**G**) (n = 6). * *p* < 0.05, ** *p* < 0.01, *** *p* < 0.001.

In addition to studying the functional impact of DARC in adipocytes in vitro, we sought to investigate the functional role of DARC in mature adipocytes in vivo. To that end, we crossed the DARC floxed mice with the adipoq-Cre mouse to specifically delete DARC in adipose tissue (Supplementary Figure S16). We employed an immunohistochemical approach to verify that DARC was indeed deleted from the adipose tissues of adipoq-Cre-expressing mice. Both subcutaneous and visceral adipose tissues of DARC floxed adipoq-Cre negative (WT) mice on CD stained positively for DARC, while adipose tissues from DARC floxed adipoq-Cre positive (DARC^Δadipo^) mice exhibited minimal DARC staining (Figure 5A). Interestingly, adipocytes from subcutaneous adipose tissues of DARC^Δadipo^ mice appeared larger compared with those from WT mice, mirroring the observations from the global DARC KO mice (Figure 3B,C). However, there was no significant difference in adipocyte size in cells derived from visceral fat (Figure 5B).

Next, we studied WT and DARC^Δadipo^ mice maintained on CD or switched to HFD at 8 weeks of age for up to 24 weeks. There was significant, but equivalent, weight gain in WT and DARC^Δadipo^ mice on HFD compared with mice maintained on CD; however, there was no difference between the genotypes (Figure 5C). Body composition measured by NMR demonstrated that DARC^Δadipo^ mice on CD had significantly greater fat mass and less lean mass compared with WT mice (Figure 5D). Differences in body composition were eliminated by HFD feeding, during which both WT and DARC^Δadipo^ mice exhibited dramatic increases in fat mass and corresponding decreases in lean mass (Figure 5D). Moreover, there was a significant and similar increase in adipose tissue weight, normalized to body weight, in both WT and DARC^Δadipo^ mice fed an HFD (Figure 5E). Additionally, there was no difference in liver weight, normalized to body weight, between WT and DARC^Δadipo^ mice on either CD or HFD (Supplementary Figure S17). Finally, GTT and ITT demonstrated impaired glucose tolerance in mice fed an HFD compared with CD; however, no differences were observed between WT and DARC^Δadipo^ mice (Figure 5F,G and Figure S4B). Taken together, these results suggest that disruption of DARC in adipocytes is associated with adipocyte enlargement and increased baseline adiposity in CD-fed mice but does not alter glucose tolerance under HFD.

### 3.5. EC-Specific Deletion of DARC Did Not Affect Body Weight or Glucose Tolerance

ECs play essential roles in angiogenesis, nutrient transport, and metabolic homeostasis in adipose tissue. DARC expressed in ECs has been reported to regulate chemokine trafficking and leukocyte extravasation, thereby contributing to vascular inflammation. To determine whether EC-derived DARC contributes to adipose tissue inflammation and glucose tolerance, we generated EC-specific DARC KO mice (DARC^ΔEC^) by crossing DARC floxed mice with VE-Cadherin-Cre mice. Consistent with findings in DARC^ΔAdipo^ mice, DARC^ΔEC^ mice fed an HFD exhibited no significant differences in body weight or glucose tolerance compared with WT controls (Supplementary Figure S18). These findings suggest that DARC expression in ECs does not significantly affect body weight and glucose tolerance.

## 4. Discussion

Adipose tissue inflammation in obesity promotes insulin resistance and metabolic disease in association with increased production of pro-inflammatory chemokines and cytokines. Here, using a novel DARC floxed mouse, we report that hematopoietic-specific disruption of DARC, a non-signaling receptor with chemokine buffering activity, enhances adipose tissue inflammation during diet-induced obesity in mice, thereby promoting glucose intolerance. These findings suggest that gene variants that impact DARC expression on erythrocytes, which are common in people of African descent, might disrupt the ability to control adipose tissue inflammation, thus predisposing to obesity-related metabolic disease and type 2 diabetes.

We previously reported that global DARC KO mice exhibited glucose intolerance in conjunction with increased adipose tissue inflammation during diet-induced obesity [12]. Levels of MCP-1, a prototypical DARC-binding chemokine, were reduced in plasma yet increased in adipose tissues of high-fat-fed DARC KO mice compared with control mice [12], consistent with impaired chemokine trafficking from inflamed adipose tissues to the erythrocyte reservoir. While such chemokine trafficking is dependent on both a chemokine gradient and DARC expression on erythrocytes, it could also involve DARC expression on other cell types, such as endothelial cells, adipocytes, etc. In this study, we report that disruption of DARC specifically in hematopoietic cells recapitulates several key findings observed with global DARC KO mice, including increased adipose tissue inflammation and altered circulating chemokine levels. The concomitant reduction in circulating DARC-binding chemokines and accumulation of inflammatory chemokines within adipose tissue is consistent with a role for DARC in regulating chemokine distribution during obesity. However, the present study does not establish that impaired erythrocyte chemokine buffering or trafficking is causally responsible for the increased adipose tissue inflammation or metabolic abnormalities. Hematopoietic-specific DARC KO mice also exhibited evidence of increased systemic oxidative stress, a finding which has been closely linked to obesity, adipose tissue inflammation, and glucose intolerance in both human and animal studies [19]. However, it is unclear why there were no differences in adipocyte number or size between WT and DARC^Δhemato^ mice fed an HFD, despite the increased adipose tissue inflammation. On the other hand, more subtle observations in the global DARC KO mouse, including increased adiposity/visceral fat mass and impaired glucose tolerance in lean male DARC KO mice, and increased weight gain during high-fat feeding, were not observed in the hematopoietic-specific DARC KO mice. These findings prompted us to examine DARC expression in non-hematopoietic tissues, and surprisingly, we detected significant DARC expression in both subcutaneous and visceral fat of wild-type mice. Additionally, DARC KO mice exhibited mild subcutaneous adipocyte hypertrophy under basal (chow-fed) conditions, and visceral adipocyte hypertrophy under HFD-fed conditions, raising the possibility that DARC expression in adipocytes could regulate their function.

To investigate this possibility, we isolated preadipocytes from DARC KO mice and investigated their capacity to differentiate into mature adipocytes and store intracellular lipids. Interestingly, DARC gene disruption did not directly affect adipogenic differentiation, lipid accumulation, or insulin-dependent glucose uptake in vitro. Using our floxed mouse, we disrupted DARC expression in mature adipocytes to investigate the function of DARC in adipocytes in vivo. Notably, the adipocyte-specific DARC KO mice exhibited subcutaneous adipocyte hypertrophy and greater fat mass under basal (chow-fed) conditions, recapitulating those findings observed in the global DARC KO mouse. However, disruption of DARC in mature adipocytes did not affect weight gain, adiposity, or glucose tolerance during HFD feeding. The reasons behind the apparently discrepant in vitro versus in vivo findings are unclear. However, it is important to point out that the in vitro adipocyte model does not perfectly replicate the process of adipose tissue development and growth at the whole tissue and animal level. In addition, the presence of subcutaneous adipocyte hypertrophy does not necessarily indicate impaired glucose tolerance, as the metabolic consequences of adipose expansion depend more on visceral fat deposition than on subcutaneous adipose tissue and its functional state, including inflammation and ectopic lipid accumulation. Thus, the preserved glucose tolerance observed in our mice despite subcutaneous adipocyte hypertrophy may reflect relatively preserved subcutaneous adipose tissue function and metabolic adaptation to HFD. Taken together, our findings suggest that expression of DARC in adipocytes may regulate some aspects of adipocyte morphology in vivo. The lack of significant metabolic differences in adipocyte-specific DARC-deficient mice suggests that loss of DARC in adipose tissue does not produce readily detectable metabolic abnormalities under the conditions examined. However, direct comparison with the hematopoietic-specific model is limited by differences in HFD exposure duration and experimental design. Moreover, the absence of statistically significant differences should not be interpreted as evidence that adipocyte DARC has no biological contribution to obesity-related metabolic regulation. Longer-term studies and experimental designs that directly compare the two models will be required to address this possibility. Nonetheless, a deeper understanding of the context- and cell type-specific functions of DARC is important and further studies will be needed to fully delineate the distinct contributions of DARC expressed in hematopoietic cells and other cell types in different metabolic and inflammatory settings.

It is important to note several limitations of the present study. First, the common DARC gene variant harbored by people of African ancestry results in selective loss of DARC expression on erythrocytes as opposed to all hematopoietic cells, which may limit the translatability of our findings. Unfortunately, to the best of our knowledge, there is no specific Cre driver that leads to selective gene disruption in erythrocytes. The erythropoietin receptor (EpoR) is an erythroid-specific gene, and EpoR-Cre mice have been used for erythrocyte-specific gene disruption. However, accumulating evidence suggests that EpoR is also expressed in other hematopoietic cells, beyond erythroid cells [21,22]. Second, DARC was reported to be expressed in a subset of macrophages and monocytes in mouse bone marrow [23]. DARC interacts with CD82 on long-term repopulating hematopoietic stem cells (LT-HSCs) to maintain the dormancy of LT-HSCs during homeostasis [24], suggesting a potential role of DARC expressed in macrophages in regulating inflammatory processes. We observed trends toward increased monocytes and neutrophils in hematopoietic-specific DARC KO mice (Supplementary Figure S6), suggesting a potential role of DARC in immune cells beyond erythrocytes. Plasma cytokine levels are also increased in hematopoietic-specific DARC KO mice (Supplementary Figure S7), which may contribute to adipose tissue inflammation. These findings are consistent with, and further support, our observation of increased macrophage infiltration (Figure 2F,G) and Mcp-1 mRNA expression (Figure 2C) in the adipose tissue of hematopoietic-specific DARC KO mice fed an HFD. However, the chemokine data generated in this study are consistent with a canonical role for DARC expression in erythrocytes serving as a “buffer-sink” to regulate inflammation. Nevertheless, further studies are warranted to investigate whether DARC expressed in monocytes and/or macrophages might play a role in obesity-related adipose tissue inflammation and metabolic disease. It is also possible that DARC expressed in other cell types, such as endothelial cells [25], could regulate chemokine trafficking in inflamed adipose tissues in obesity. Thus, the relatively modest phenotype observed in adipocyte-specific DARC KO mice should be interpreted with caution because DARC expression was assessed in whole adipose tissue rather than separately in mature adipocytes and stromal vascular fraction cells. Notably, endothelial cell-specific DARC deficiency resulted in adipose tissue and metabolic phenotypes broadly similar to those observed in adipocyte-specific DARC KO mice, suggesting a minimal role of DARC expressed by non-adipocyte populations in adipose tissue inflammation and metabolic dysfunction. Further studies using isolated mature adipocytes and stromal vascular fraction cells will be necessary to define the cell-specific contribution of DARC within adipose tissue. Third, we unexpectedly did not observe significant liver weight gain and adipocyte hypertrophy in HFD-fed WT mice. Previous studies, including our own, have shown that mice housed at standard ambient temperature (20–22 °C), as opposed to thermoneutral conditions (28–30 °C), exhibit increased energy expenditure, reduced adipocyte hypertrophy and relative resistance to diet-induced obesity [14,26]. All mice in this study were maintained at ambient temperature, which may have attenuated changes in liver weight and adipocyte size. Fourth, although GTT and ITT data showed significant differences between WT and DARC-deficient mice, hyperinsulinemic-euglycemic clamp studies would provide additional quantitative information on tissue-specific insulin sensitivity. In addition, GTT and ITT were performed at different time points in the hematopoietic- and adipocyte-specific KO mice (20–24 weeks vs. 40 weeks of age), which may limit direct comparisons between these groups. Moreover, metabolic cage analyses were not performed to assess food intake, energy expenditure, or physical activity, which could influence metabolic status. Fifth, we did not perform investigations on female mice in this study. However, in our prior study, we observed reduced glucose tolerance in high-fat-fed female global DARC KO mice, suggesting that the principal findings reported here in hematopoietic-specific DARC KO male mice fed an HFD are unlikely to be sex-specific. Future studies are required to confirm the findings of this study in female mice. Finally, several mechanistic analyses, including adipose tissue chemokine and macrophage-associated gene expression measurements, were performed with relatively small sample sizes (n = 3), which limits the statistical power and generalizability of these findings. These data should, therefore, be considered supportive rather than definitive evidence of the proposed mechanism.

Hematopoietic-specific disruption of DARC led to liver enlargement and hepatic steatosis in HFD-fed male mice, a finding that was also observed in global DARC KO male mice [12]. Steatosis, or ectopic lipid deposition in the liver, is closely linked to the development of obesity, worsening metabolic syndrome, and adipose tissue-derived inflammation [27]. The mechanisms responsible for these findings are unclear. However, in our prior study, we did not detect liver enlargement in global DARC KO female mice fed an HFD [12]. This suggests that the chemokine imbalance resulting from disruption of DARC during obesity, in itself, is insufficient to promote hepatic steatosis, implying a possible sex-specific modifying effect. We did not investigate the mechanisms of the intrahepatic hematopoietic system, such as hepatic stem cells, in this study. Furthermore, although we showed no difference in plasma levels of AST and triglycerides, these cannot be used as a direct surrogate for intrahepatic triglyceride accumulation and future studies directly measuring hepatic triglyceride content will be needed to further characterize the effect of hematopoietic DARC deficiency on hepatic lipid accumulation.

In conclusion, our data suggest that disruption of DARC in hematopoietic cells is associated with altered circulating chemokine levels, increased adipose tissue inflammation and oxidative stress during obesity and impaired glucose homeostasis during diet-induced obesity. Given the differences between the hematopoietic-specific mouse model used here and the erythrocyte-selective DARC deficiency associated with human genetic variants, further studies are required to determine the relevance of these findings to human DARC variation and metabolic disease.

## Data Availability

The raw data supporting the conclusions of this article will be made available by the authors on request.

## Supplementary Materials

The following supporting information can be downloaded at: https://www.mdpi.com/article/10.3390/cells15181664/s1, Figure S1. Construction and validation of hematopoietic-specific DARC KO mouse. (A) DARC (Ackr1) flox allele map. (B) Flow cytometry of RBCs obtained from WT, global DARC KO, DARC flox/Vav1Cre- and DARC flox/Vav1Cre+ mice. (C) mRNA expression of DARC expression in BM, spleen and subcutaneous adipose tissue from the four groups of mice (n = 3–5). **** *p* < 0.0001. two-way ANOVA. Figure S2. Plasma levels of AST in WT and DARC^Δhemato^ mice (n = 4–6). Figure S3. Plasma levels of cholesterol and triglycerides in WT and DARC^Δhemato^ mice on either CD or HFD (n = 3). * *p* < 0.05, ** *p* < 0.01. Figure S4. Area under curve (AUC) for GTT data and the constant for plasma glucose disappearance (KITT) for ITT data. ((A) Figure 1F,G, (B) Figure 5F,G). Figure S5. Fasting plasma insulin levels measured by ELISA in WT and DARC^Δhemato^ mice on either CD or HFD. the CD and HFD groups (n = 4). **** *p* < 0.0001. Figure S6. Phosphorylation of Akt normalized by total AKT in adipose tissues, skeletal muscles and liver of WT and DARC^Δhemato^ mice. Figure S7. Percentage of monocytes, neutrophils, conventional dendritic cells (cDCs) and natural killer (NK) cells in peripheral blood as assessed by flow cytometry. * *p* < 0.05. Figure S8. Plasma levels of pro-inflammatory cytokines in WT and DARCΔhemato mice (n = 4–6). * *p* < 0.05, ** *p* < 0.01. Figure S9. Plasma levels of chemokines in WT and DARCΔhemato mice (n = 3–6). * *p* < 0.05. Figure S10. mRNA expression of CCL5 and CXCL1 in adipose tissues of WT and DARCΔhemato mice (n = 3). * *p* < 0.05. Figure S11. mRNA expression of M1 and M2 marker genes in adipose tissues of WT and DARCΔhemato mice (n = 3). * *p* < 0.05, ** *p* < 0.01. Figure S12. No difference in adipocyte number or size between WT and DARCΔhemato mice fed a HFD. Adipocyte count ((A) 121.9 ± 5.368 vs. 110.0 ± 6.988 per high power field 400×) and adipocyte size (B) were quantified using Adiposoft plugin for Fiji. Figure S13. Negative controls for DARC immunostaining. Figure S14. Adiponectin (A,C) and C/EBPα (B,D) before (day 0) and after adipogenic differentiation (day 14, n = 4). ** *p* < 0.01, *** *p* < 0.001. Figure S15. Loss of DARC does not impair insulin stimulated glucose uptake or AKT phosphorylation in adipocytes in vitro. (A) Preadipocytes isolated from SQ adipose tissues were differentiated into mature adipocytes for 14 days and glucose uptake was measured (n = 3). (B) Levels of AKT phosphorylation in mature adipocytes with and without insulin treatment (n = 3). * *p* < 0.05. Figure S16. Generation of an adipose specific DARC knockout mouse. Validation of the presence of the WT/floxed DARC allele (top) and/or the adipoq-Cre allele using PCR-based genotyping. Figure S17. No difference in liver weight, normalized to body weight, between WT and DARC^Δadipo^ mice on either CD or HFD (n = 3–5). Figure S18. (A) Growth curves of WT and DARCΔEC mice fed HFD (n = 6). (B,C) GTT after 20 weeks HFD (B) and ITT after 21 weeks HFD (C) (n = 6). Table S1. Primer sequences.
