# Supplementary material for: Hematopoietic-Specific DARC Deficiency Is Associated with Adipose Tissue Inflammation and Impaired Glucose Tolerance During Diet-Induced Obesity in Mice
Source: Cells. 2026 Sep 15;15(18):1664. doi: 10.3390/cells15181664 (PMC13606003; doi:10.3390/cells15181664)
Supplement: Supplementary file 1 [file cells-15-01664-s001.zip › cells-4459328-supplementary.pdf]

Supplemental material

Supplemental Figure S1

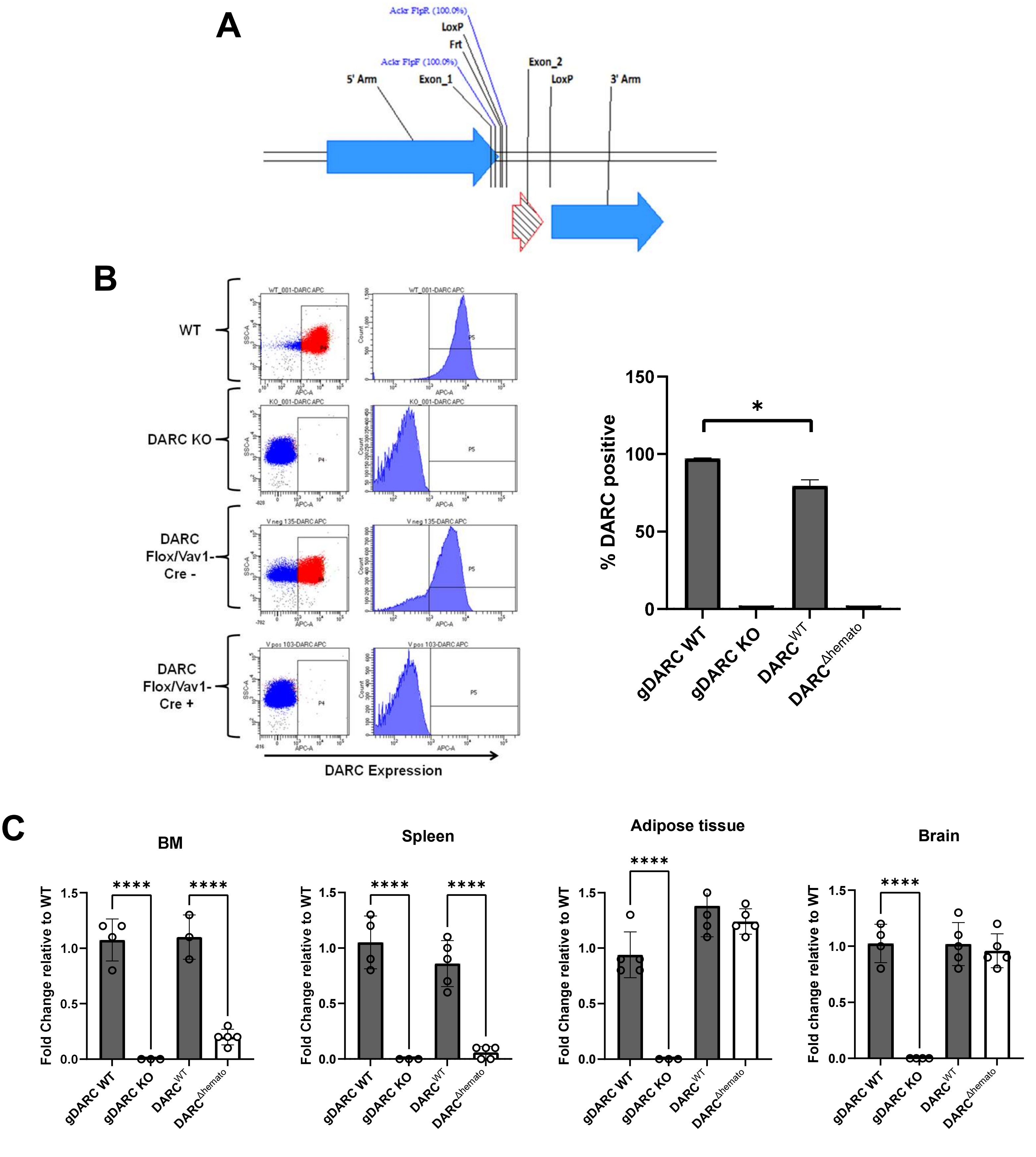

Supplemental Figure S1. Construction and validation of hematopoietic-specific DARC KO mouse. (A) DARC (Ackr1) flox allele map. (B) Flow cytometry of RBCs obtained from WT, global DARC KO, DARC flox/Vav1Cre- and DARC flox/Vav1Cre+ mice. (C) mRNA expression of DARC expression in BM, spleen and subcutaneous adipose tissue from the four groups of mice (n=3-5). ****p < 0.0001. two-way ANOVA.

Supplemental Figure S2

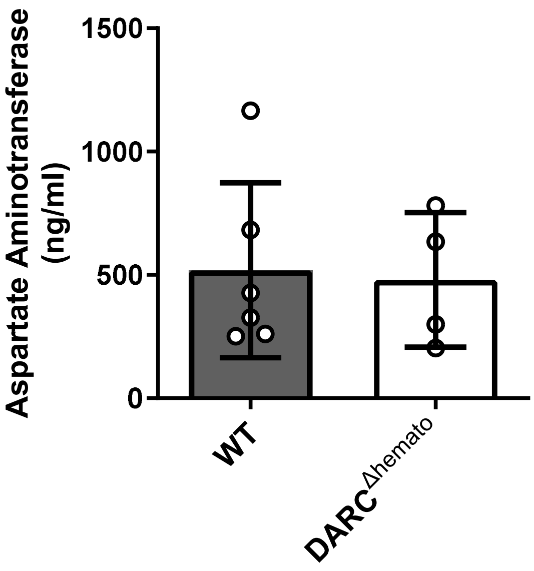

Supplemental Figure S2. Plasma levels of AST in WT and DARC$^{\Delta hemato}$ mice (n=4-6).

Supplemental Figure S3

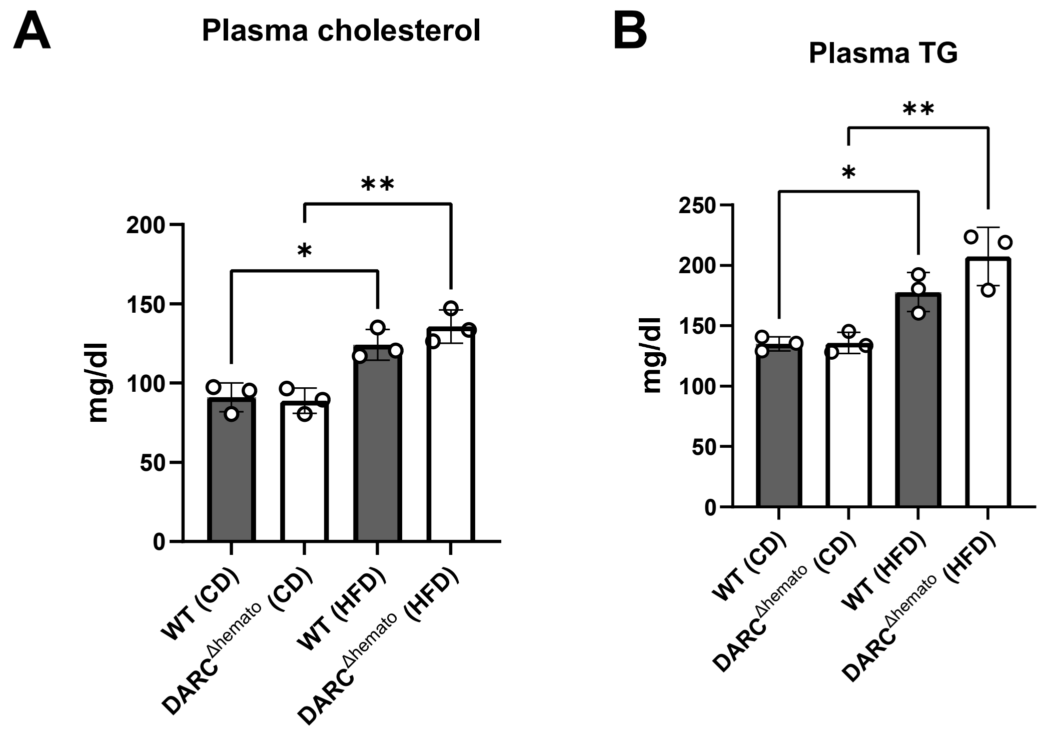

Supplemental Figure S3. Plasma levels of cholesterol and triglycerides in WT and DARC$^{\Delta hemato}$ mice on either CD or HFD (n=3). *$p < 0.05$, **$p < 0.01$.

Supplemental Figure S4

A

WT vs DARC$^{\Delta hemato}$ mice

	WT (HFD)	KO (HFD)	p	WT (CD)	KO (CD)	p
**GTT AUC**	40568 ± 3315	51169 ± 1533	0.0272	30885 ± 875	32692 ± 3084	0.5933
**KITT (%/MIN)**	0.439 ± 0.203	0.272 ± 0.132	0.5165	0.331 ± 0.071	0.179 ± 0.120	0.3173

B

WT vs DARC$^{\Delta Adipo}$ mice

	WT (HFD)	KO (HFD)	p	WT (CD)	KO (CD)	p
**GTT AUC**	40311 ± 1986	36322 ± 2375	0.2350	25448 ± 2201	25340 ± 1436	0.9715
**KITT (%/MIN)**	0.245 ± 0.091	0.440 ± 0.096	0.5006	0.635 ± 0.077	0.680 ± 0.044	0.6674

Supplemental Figure S4. Area under curve (AUC) for GTT data and the constant for plasma glucose disappearance (KITT) for ITT data. (A: Figure 1F&G, B: Figure 5F&G).

Supplemental Figure S5

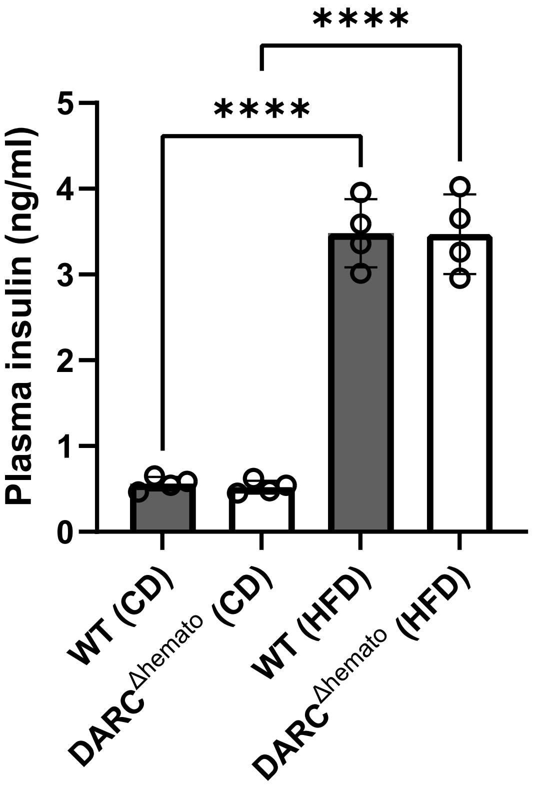

Supplemental Figure S5. Fasting plasma insulin levels measured by ELISA in WT and DARC$^{\Delta hemato}$ mice on either CD or HFD. the CD and HFD groups (n = 4). ****$p < 0.0001$.

Supplemental Figure S6

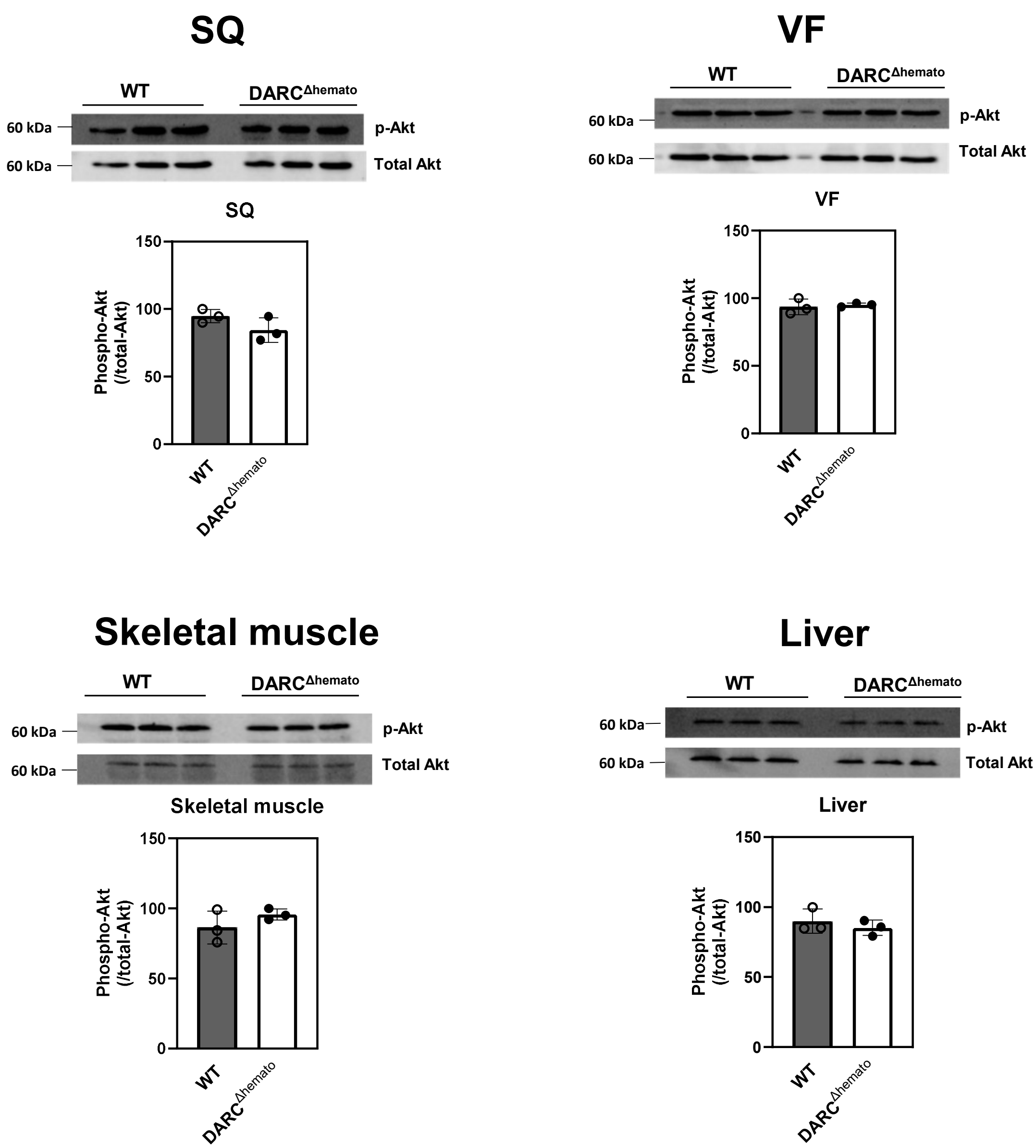

Supplemental Figure S6. Phosphorylation of Akt normalized by total AKT in adipose tissues, skeletal muscles and liver of WT and DARC$^{\Delta hemato}$ mice.

Supplemental Figure S7

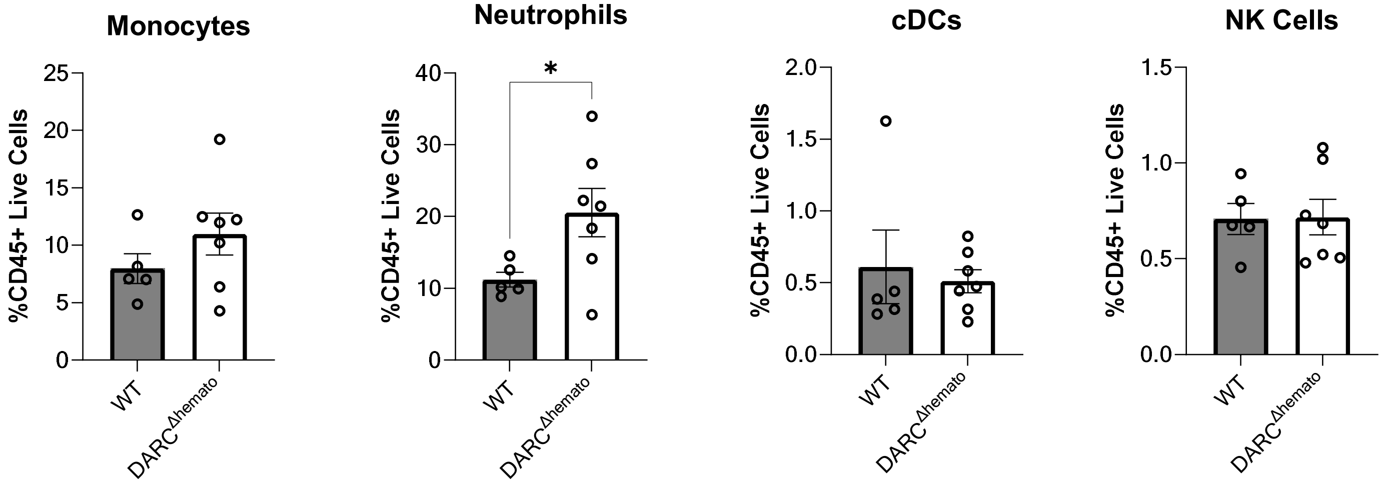

Supplemental Figure S7. Percentage of monocytes, neutrophils, conventional dendritic cells (cDCs) and natural killer (NK) cells in peripheral blood as assessed by flow cytometry. *p < 0.05.

Supplemental Figure S8

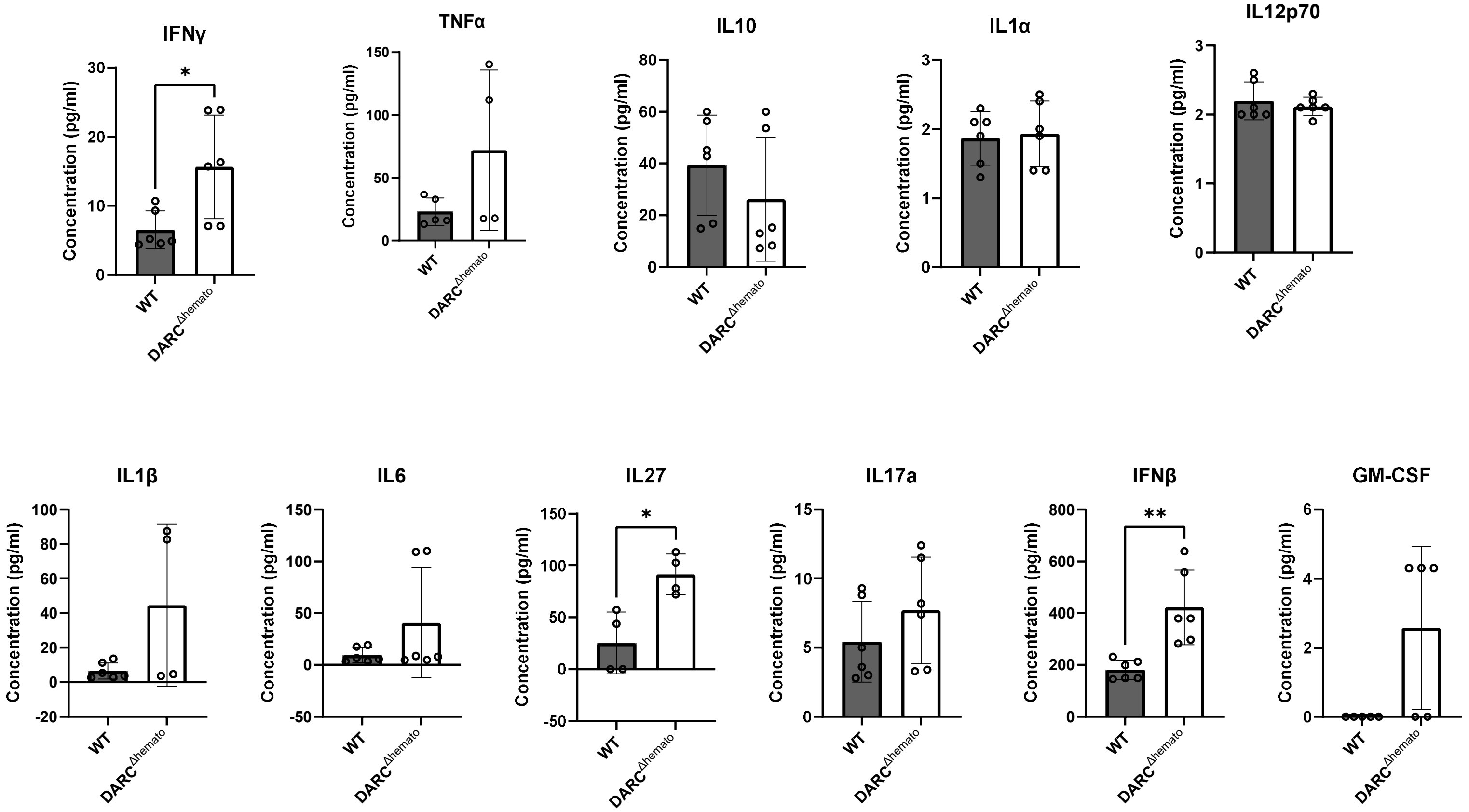

Supplemental Figure S8. Plasma levels of pro-inflammatory cytokines in WT and DARC$^{\Delta hemato}$ mice (n=4-6). *$p < 0.05$, **$p < 0.01$.

Supplemental Figure S9

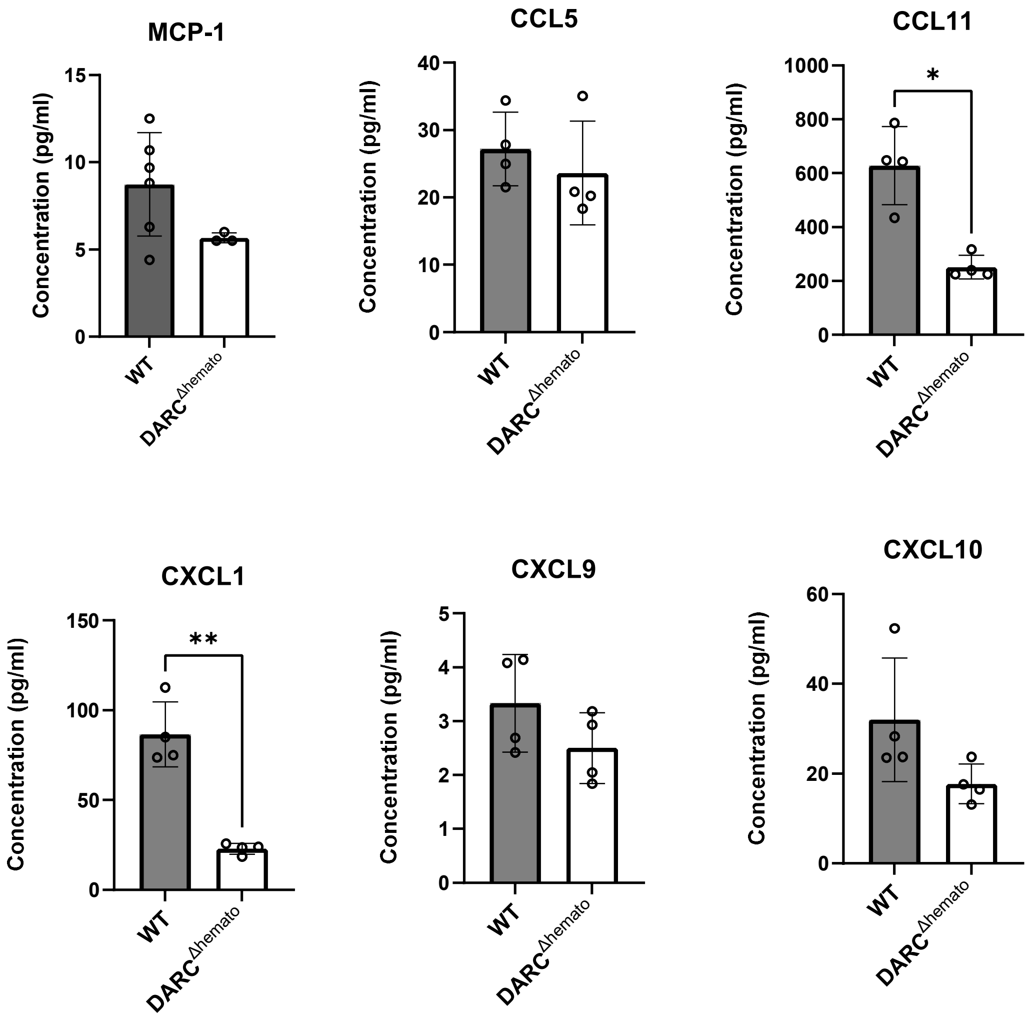

Supplemental Figure S9. Plasma levels of chemokines in WT and DARC$^{\Delta hemato}$ mice (n=3-6). *$p < 0.05$.

Supplemental Figure S10

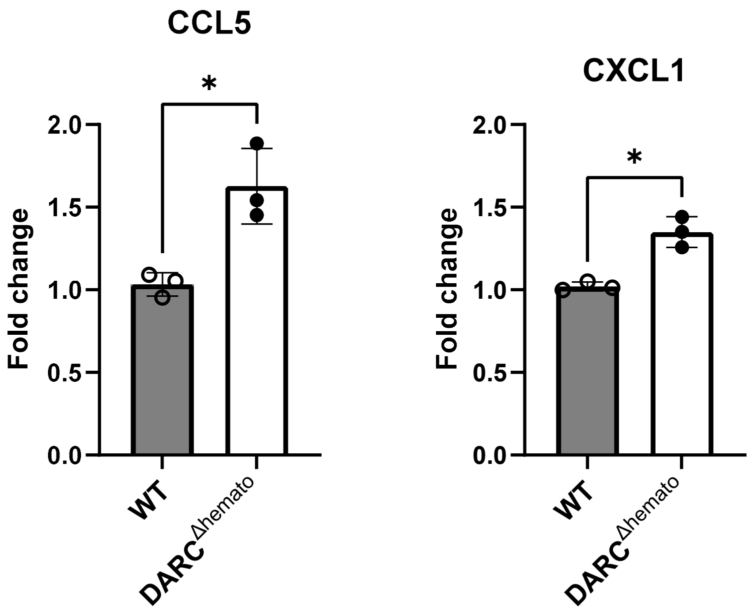

Supplemental Figure S10. mRNA expression of CCL5 and CXCL1 in adipose tissues of WT and DARC$^{\Delta hemato}$ mice (n=3). *$p < 0.05$.

Supplemental Figure S11

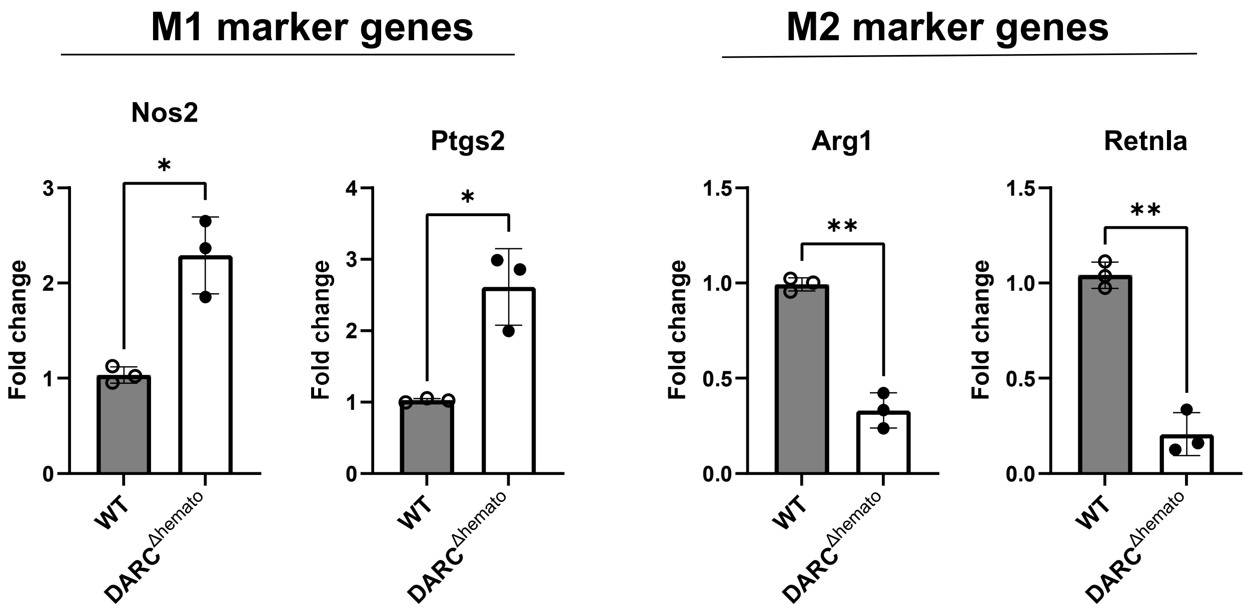

Supplemental Figure S11. mRNA expression of M1 and M2 marker genes in adipose tissues of WT and DARC$^{\Delta hemato}$ mice (n=3). *p < 0.05, **p < 0.01.

Supplemental Figure S12

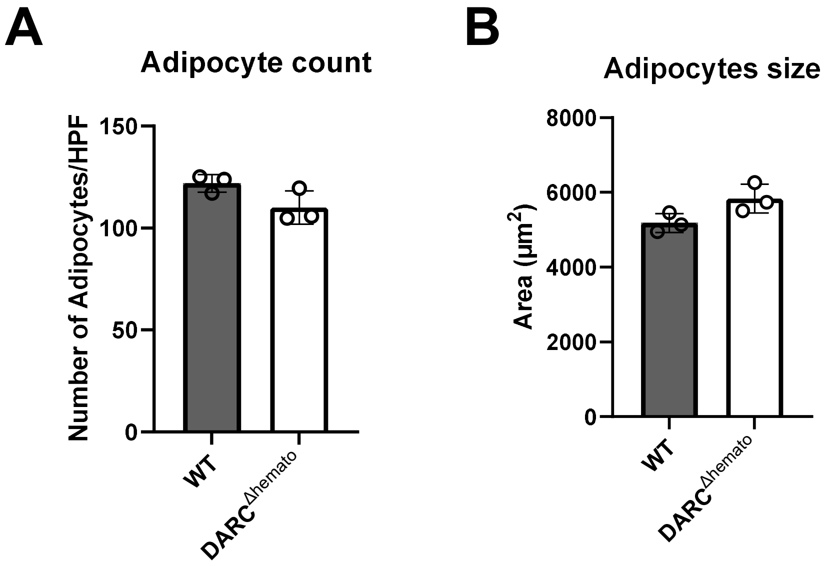

Supplemental Figure S12. No difference in adipocyte number or size between WT and DARC$^{\Delta hemato}$ mice fed a HFD. Adipocyte count (A: 121.9±5.368 vs 110.0±6.988 per high power field 400x) and adipocyte size (B) were quantified using Adiposoft plugin for Fiji.

Supplemental Figure S13

Negative controls for DARC immunostaining

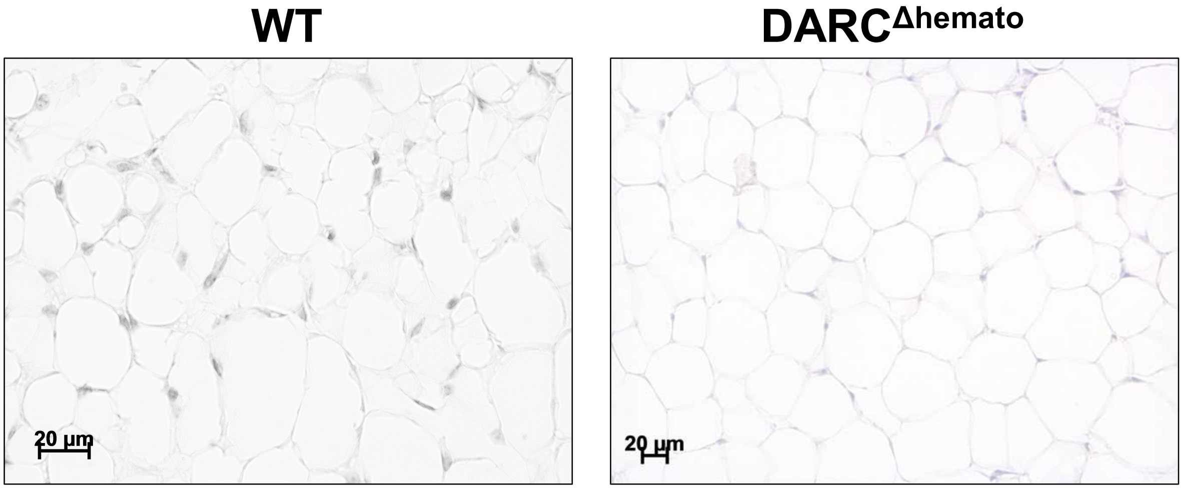

Supplemental Figure S14

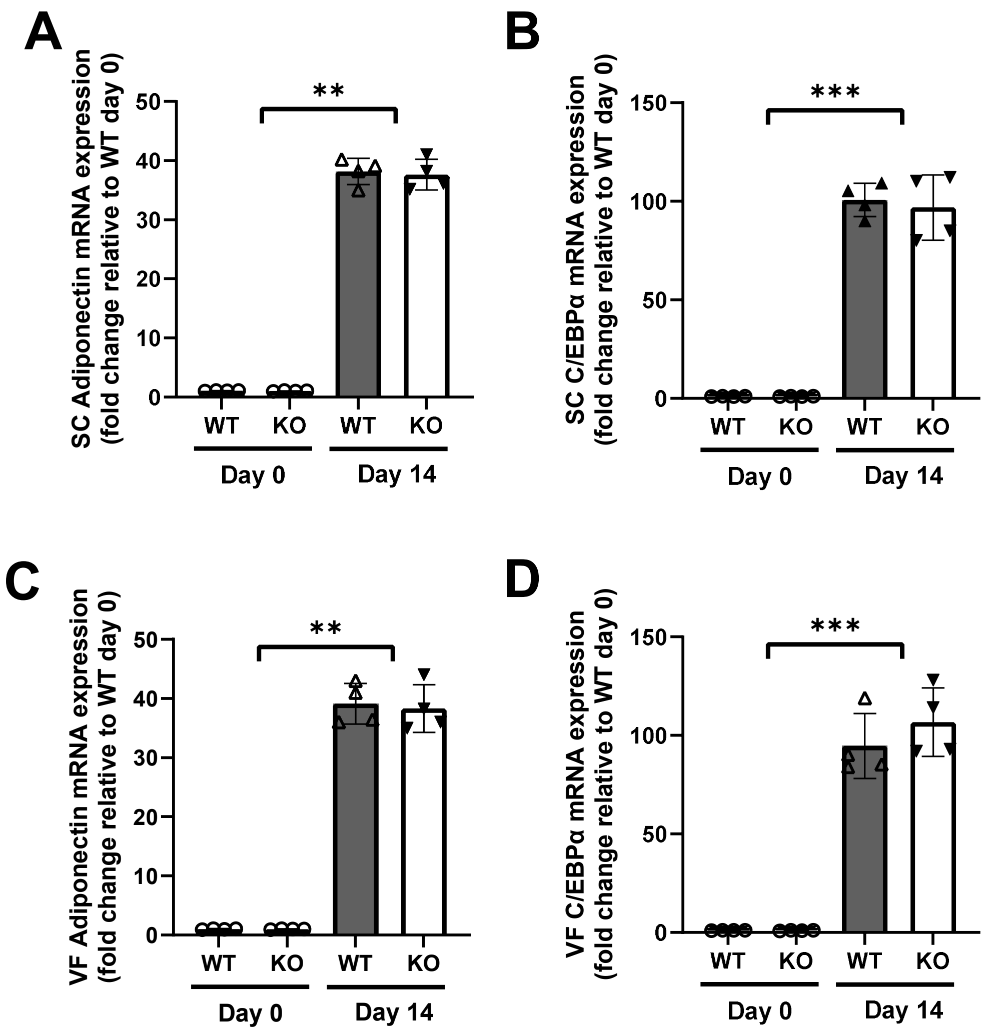

Supplemental Figure S14. Adiponectin (**A, C**) and C/EBPα (**B, D**) before (day 0) and after adipogenic differentiation (day 14, n=4). **p < 0.01, ***p < 0.001.

Supplemental Figure S15

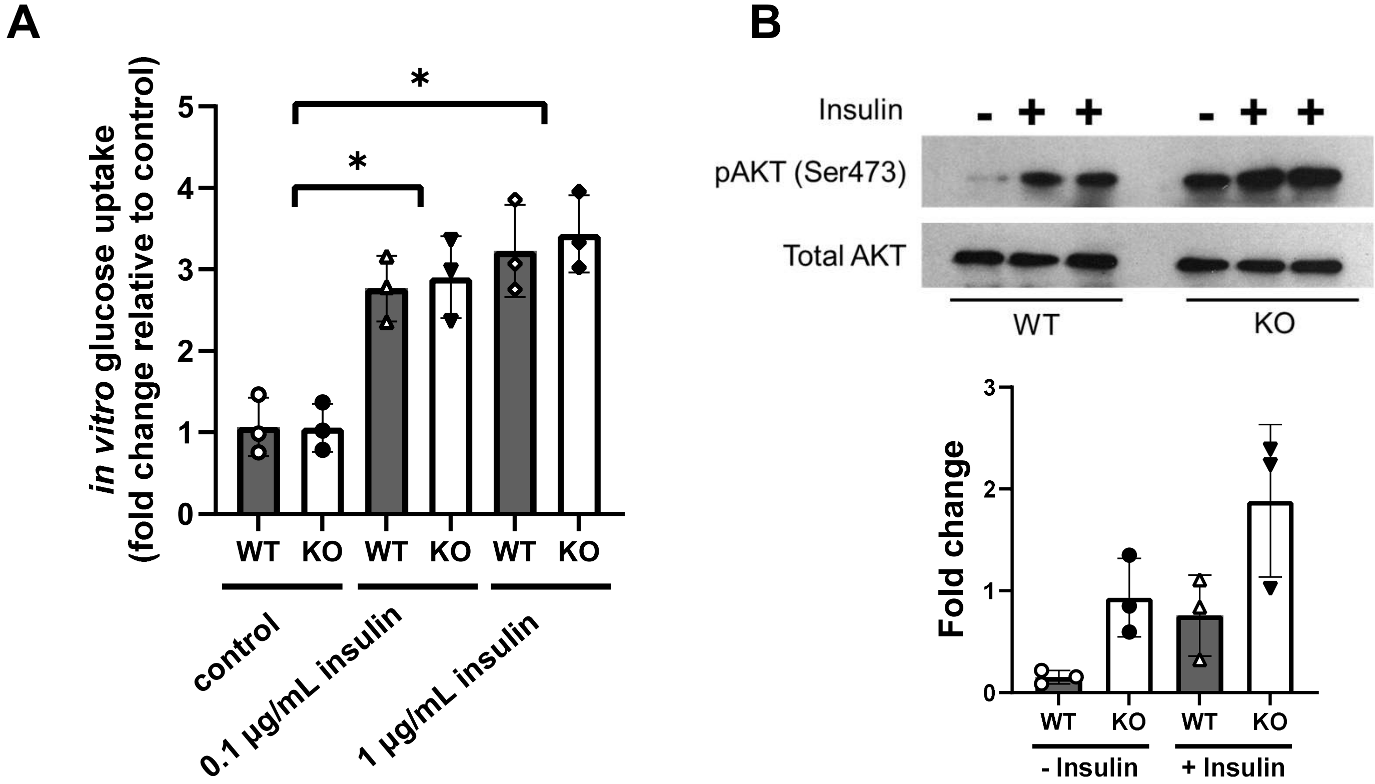

Supplemental Figure S15. Loss of DARC does not impair insulin stimulated glucose uptake or AKT phosphorylation in adipocytes *in vitro.* (A) Preadipocytes isolated from SQ adipose tissues were differentiated into mature adipocytes for 14 days and glucose uptake was measured (n=3). (B) Levels of AKT phosphorylation in mature adipocytes with and without insulin treatment (n=3). **p*<0.05.

Supplemental Figure S16

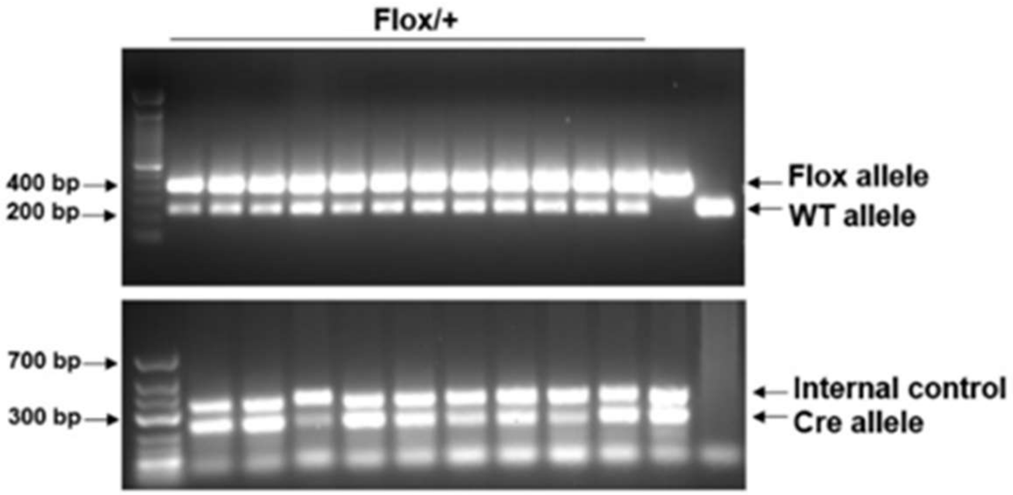

Supplemental Figure S16. Generation of an adipose specific DARC knockout mouse. Validation of the presence of the WT/floxed DARC allele (top) and/or the adipoq-Cre allele using PCR-based genotyping.

Supplemental Figure S17

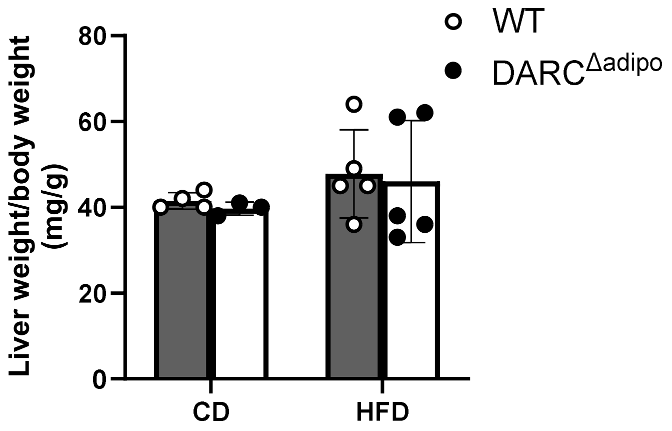

Supplemental Figure S17. No difference in liver weight, normalized to body weight, between WT and DARC$^{\Delta adipo}$ mice on either CD or HFD (n=3-5).

Supplemental Figure S18

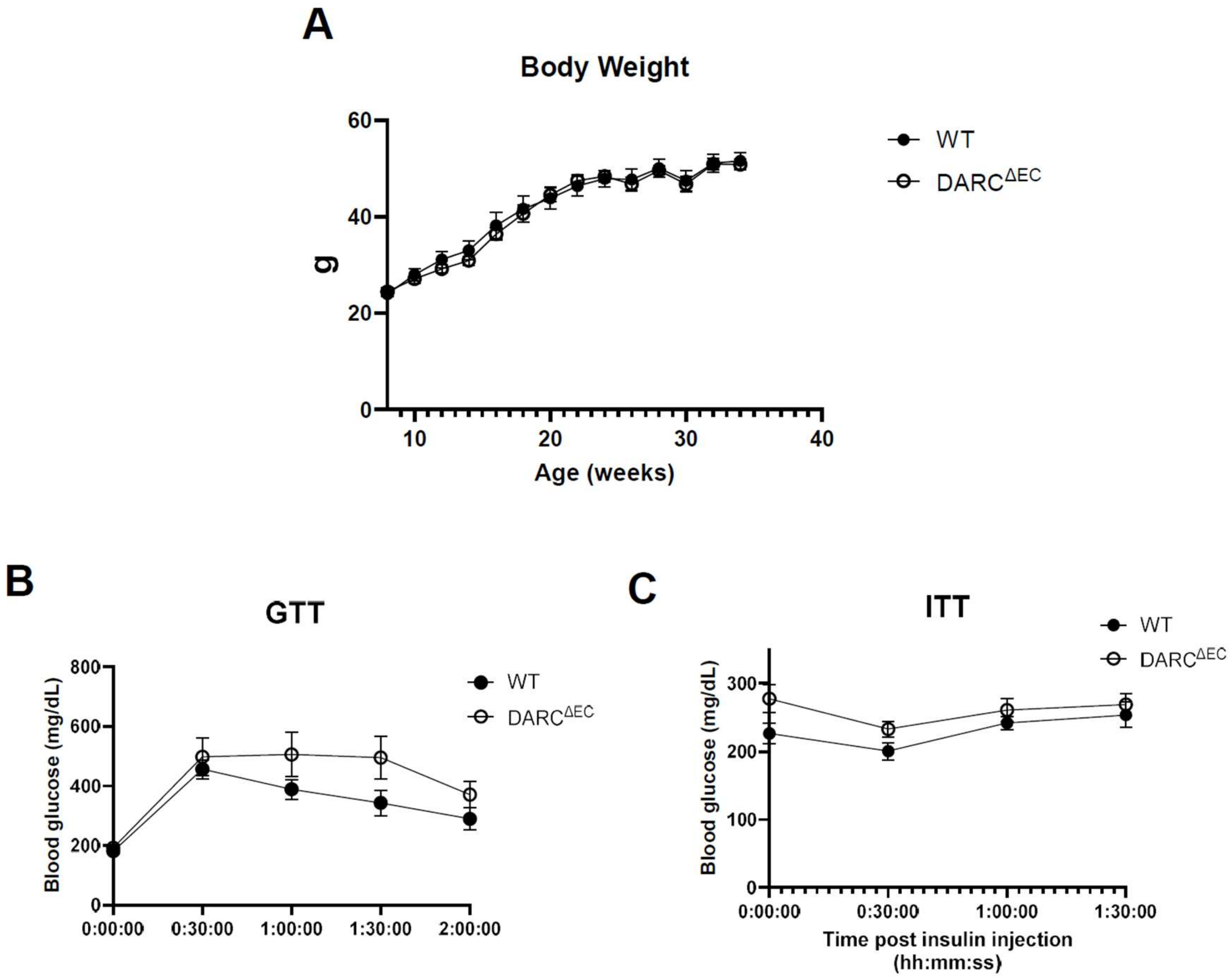

Supplemental Figure S18. (A) Growth curves of WT and DARCΔEC mice fed HFD (n=6). (B,C) GTT after 20 weeks HFD (B) and ITT after 21 weeks HFD (C) (n=6).

Supplemental Table S1

Primer sequences:

•*Arbp*
- Forward: 5’-AGC TGA AGC AAA GGA AGA GTC GGA-3’
- Reverse: 5’-ACT TGG TTG CTT TGG CGG GAT TAG-3’

•*Darc*
- Forward: 5’-TCC GGT GGA AAA CCT TTC ACT A-3’
- Reverse: 5’-GCT GGT GTC AGG CTG TAG TC-3’

•*Mcp-1*
- Forward: 5’-GGC TCA AGC CAG ATG CAG TTA C-3’
- Reverse: 5’-GCC TAC TCA TTG GGA TCA TCT T-3’

•*Il-6*
- Forward: 5’-CTT CCA TCC AGT TGC CTT CT-3’
- Reverse: 5’-CTC CG ACTT GTG AAG TGG TAT AG-3’

•*Tnfα*
- Forward: 5’-TCT CAT GCA CCA CCA TCA AGG ACT-3’
- Reverse: 5’-ACC ACT CTC CCT TTG CAG AAC TCA-3’

•*Pparγ*
- Forward: 5'-ACA TAA AGT CCT TCC CGC TGA CCA-3'
- Reverse: 5'-AAA TTC GGA TGG CCA CCT CTT TGC-5’

•*Fabp4*
- Forward: 5'-ATG AAA TCA CCG CAG ACG ACA GGA-3'
- Reverse: 5'-TGT GGT CGA CTT TCC ATC CCA CTT-3'
